# Genome-Wide Characterization and Multidimensional Transcriptome Profiling of the SABATH Methyltransferase Gene Family in Sunflower (*Helianthus annuus* L.)

**DOI:** 10.3390/ijms27219805

**Published:** 2026-10-31

**Authors:** Xinlong Gao, Kaihui Wang, Mengzhe Li, Dan Li, Xu Liu, Jiangna Zheng, Suen Liu, Junyi Geng, Baosheng Guo, Yang Han, Haina Zhang, Cunpeng Zhao

**Affiliations:** Institute of Cotton, Hebei Academy of Agriculture and Forestry Sciences, Key Laboratory of Cotton Biology and Genetic Breeding in Huanghuaihai Semiarid Area, Ministry of Agriculture and Rural Affairs, Hebei Key Laboratory of Cotton Bio-Breeding and Cultivation Physiology, Shijiazhuang 050051, China; gaoxl4work@gmail.com (X.G.);

**Keywords:** *Helianthus annuus*, SABATH methyltransferase, gene duplication, phylogeny, synteny, transcriptome profiling

## Abstract

Plant SABATH methyltransferases contribute to volatile formation, hormone homeostasis and stress responses, but their family-wide organization in sunflower remains poorly characterized. Using the HanXRQr2.0-SUNRISE reference genome, we identified 33 high-confidence *HaSABATH* genes and integrated phylogenetic, duplication, synteny, promoter and protein-structure analyses with expression profiles from 200 public RNA sequencing (RNA-seq) libraries representing 87 distinct conditions. Twelve genes belonged to the salicylic acid/jasmonic acid methyltransferase-related Clade I, 19 to the benzoic acid/salicylic acid methyltransferase-related Clade II, and two to other lineages. Tandem, proximal, dispersed and whole-genome/segmental duplication contributed to the family, with overlapping tandem and segmental evidence for *HaSABATH4* and *HaSABATH15*. Eleven reliable nonsynonymous-to-synonymous substitution ratios were below one. Most predicted proteins showed high model confidence. Across the sampled conditions, Clade I members generally showed higher expression levels and broader expression, while many Clade II members were weakly expressed or condition-biased. *HaSABATH4* and *HaSABATH6* were prioritized for follow-up under combined saline–alkali stress. This integrated analysis links evolutionary context to expression divergence and provides candidates for targeted biochemical and genetic studies.

## 1. Introduction

Sunflower (*Helianthus annuus* L.) is an important oilseed and ornamental crop whose yield and quality can be affected by abiotic stresses, including drought and saline–alkali stress, and by biotic stresses, including *Sclerotinia sclerotiorum* infection and *Orobanche cumana* parasitism [1,2,3,4,5]. A previous study of combined saline–alkali stress reported genotype-associated expression of a sunflower locus annotated as benzoic acid carboxyl methyltransferase (BAMT), with its expression trend supported by quantitative reverse-transcription PCR (qRT-PCR) [6]. However, an integrated understanding of lineage composition, duplication history and expression remains lacking for the SABATH small-molecule methyltransferase family to which this locus belongs. The similarities and differences in expression among related members across organs and treatment contexts remain to be clarified.

The SABATH family encodes a group of plant small-molecule methyltransferases whose name was coined from salicylic acid methyltransferase (SAMT), benzoic acid methyltransferase (BAMT) and theobromine synthase [7,8,9,10]. SABATH proteins use S-adenosyl-L-methionine (SAM) as the methyl donor and catalyze carboxyl or N-methylation of plant hormones and secondary metabolites, including salicylic acid, jasmonic acid, indole-3-acetic acid, benzoic acid and xanthine alkaloids [10,11,12,13,14,15]. BAMT and benzoic acid/salicylic acid carboxyl methyltransferase (BSMT) catalyze the formation of methyl benzoate or methyl salicylate, which are associated with floral scent, insect interactions and defense signaling [9,16,17,18]. AmBAMT from snapdragon (*Antirrhinum majus*) was one of the earliest purified plant BAMT enzymes and catalyzes methyl benzoate formation [9], whereas *Arabidopsis* AtBSMT1 (AT3G11480) can methylate benzoic acid and salicylic acid [17]. A characterized *Arabidopsis* indole-3-acetic acid methyltransferase participates in the regulation of leaf development, and gibberellin methyltransferases may contribute to gibberellin inactivation [14,15].

Substrate recognition by SABATH members is related to their molecular structure and evolutionary background. Crystal-structure studies indicate that substrate-pocket shape and charge contribute to substrate recognition [12,19], and experimental work in *Populus* has shown that a single amino-acid substitution can switch substrate preference in a SABATH enzyme [20]. Within a phylogenetic framework of 1578 SABATH sequences, Dubs et al. used enzymatic comparisons to show that the preference of SAMT orthologs for salicylic acid over benzoic acid was generally conserved, while also observing substrate-preference shifts that may be associated with gene duplication [21]. The discovery of anthranilic acid methyltransferase activity in distinct SABATH backgrounds further suggests that similar methylation functions can be acquired independently [22]. These studies provide a basis for comparing functional conservation and divergence among family members, whereas the specific substrates and physiological roles of sunflower members still require corresponding functional evidence.

Genome-wide SABATH studies have identified 24 genes in *Arabidopsis* and 21 in rice [23], 30 in *Salvia miltiorrhiza* [24], 28 in soybean [25], 37 in upland cotton [26], 32 in tea plants [27] and 18 proteins in common bean [28]. Tandem and segmental duplication contributed to SABATH family expansion in *Arabidopsis* and rice [23], whereas expansion in *Populus* was mainly associated with tandem duplication; its members differed in tissue expression, and some proteins also showed different substrate preferences [20]. Members in *Salvia miltiorrhiza* and tomato show different organ-expression biases [24,29], and tea plant members exhibit different expression changes and temporal patterns in response to hormone treatments and tea geometrid feeding [27]. The sunflower lineage has also undergone whole-genome duplication [30], but the duplication origins and lineage distribution of its SABATH members remain to be clarified.

The same SABATH member can show organ-biased expression and respond to hormone treatment. The *Arabidopsis AtBSMT1* gene is highly expressed in flowers, and its leaf expression can be induced by methyl jasmonate, although the encoded enzyme uses salicylic acid and benzoic acid as substrates [17]. Thus, the hormone inducing expression is not necessarily a substrate of the enzyme. Studies of alkaline stress, broomrape interactions and *Sclerotinia* infection in sunflower have also observed organ- or resistance-associated differences in transcriptional responses [3,4,5]. For the reported BAMT locus [6] and its related members, differences between genotypes before combined saline–alkali treatment need to be distinguished from subsequent temporal changes, and the direction and timing of these changes need to be compared among members. Their expression ranges across other organs, hormone treatments and stress contexts also remain to be defined.

Here, we used the HanXRQr2.0-SUNRISE genome to identify and analyze the sunflower SABATH family and combined multispecies phylogeny, genomic duplication relationships and multicondition expression profiles to compare divergence among lineages and related members. We addressed three exploratory research questions: what is the lineage composition of the sunflower SABATH family; how have different duplication modes contributed to family expansion; and how do members from different lineages and duplication backgrounds differ in expression across tissues, hormone treatments and stress conditions, and which candidate genes can be prioritized on this basis? The integrated analysis reveals expression differences and condition biases among lineages and shows different stress-associated expression timing among related members with different duplication backgrounds, providing candidates for subsequent functional comparisons.

## 2. Results

### 2.1. Genome-Wide Identification and Basic Features

PF03492 hidden Markov model (HMM) searching and BLASTP validation identified 33 high-confidence *HaSABATH* genes. These genes were named *HaSABATH1* to *HaSABATH33* according to chromosomal position (Table 1). The raw HMMER output also contained one partial candidate, *LOC110900893*, whose coverage of the PF03492 HMM model coordinates was 64.2%, below the 70% screening threshold. It was therefore excluded from the 33-member set, was not assigned a *HaSABATH* name and was not used in downstream analyses; its National Center for Biotechnology Information (NCBI) annotation as “LOW QUALITY PROTEIN: jasmonate O-methyltransferase” provided consistent auxiliary quality-control information.

The 33 HaSABATH proteins ranged from 352 to 459 amino acids (aa) in length, with a mean of 370.8 aa. Coverage of the PF03492 HMM model coordinates ranged from 92.8% to 99.7%. Coding sequence (CDS) lengths ranged from 1059 to 1380 bp, molecular weights from 39.75 to 52.09 kDa and isoelectric point (pI) values from 5.12 to 8.11. All grand average of hydropathicity (GRAVY) values were negative (−0.434 to −0.100), indicating that HaSABATH proteins are generally hydrophilic.

BLASTP (NCBI BLAST+ v2.12.0) best-hit analysis showed that 26 of the 33 HaSABATH proteins (78.8%) had their best *Arabidopsis* matches within the BAMT/BSMT-related lineage of Clade II in this study, including functionally specialized members within this lineage. Fifteen matched AT5G66430 in the *Arabidopsis* BSMT-related branch, five matched AT3G11480 (BSMT1), four matched AT5G38020 (BSMT-like) and two matched AT5G04370 (nicotinic acid methyltransferase 1, NAMT1). This result indicates a preponderance of matches to reference proteins in the BAMT/BSMT-related lineage at the BLASTP best-hit level.

### 2.2. Phylogenetic Analysis and Lineage Classification

**Figure 1:**
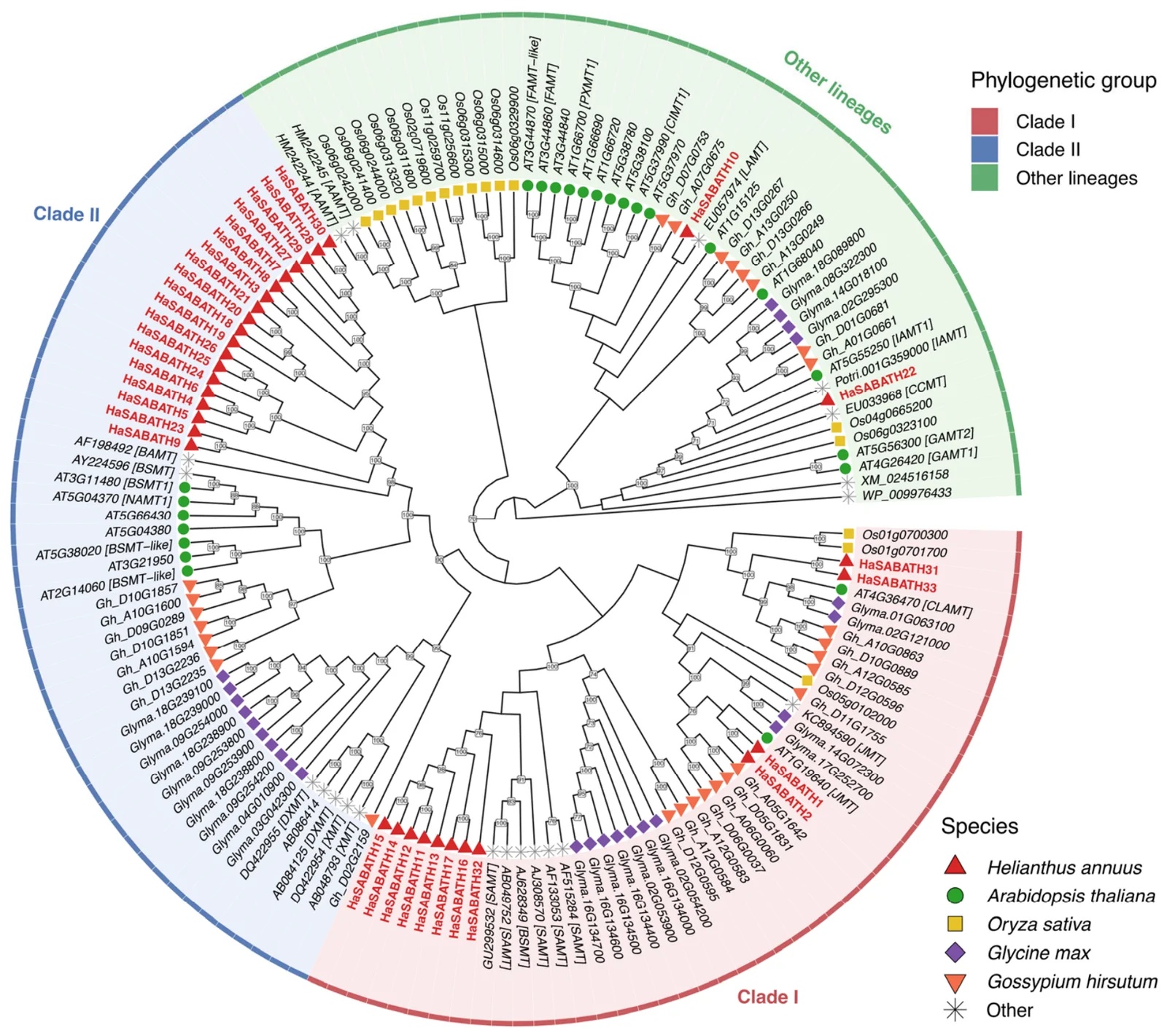
**Phylogenetic classification of sunflower and reference SABATH proteins.** The maximum-likelihood tree contains 33 HaSABATH proteins and 115 reference sequences and is rooted with WP_009976433 (*Mycobacterium avium*, MaSABATH) as the outgroup. Background colors denote Clade I (SAMT/JMT-related), Clade II (BAMT/BSMT-related) and Other lineages. Bold red labels indicate the HaSABATH members identified in this study. Ultrafast bootstrap (UFBoot) values are shown for nodes with support ≥ 70. The complete tree with full tip labels is provided in Figure S1. SAMT, salicylic acid carboxyl methyltransferase; JMT, jasmonic acid carboxyl methyltransferase; BAMT, benzoic acid carboxyl methyltransferase; BSMT, benzoic acid/salicylic acid carboxyl methyltransferase.

The multispecies maximum-likelihood (ML) tree of 148 sequences resolved two principal phylogenetic clades and a residual group referred to here as Other lineages (Figure 1). Clade I, related to SAMT and jasmonic acid carboxyl methyltransferase (JMT), contained 47 sequences, including 12 HaSABATH proteins (HaSABATH1, HaSABATH2, HaSABATH11–HaSABATH17 and HaSABATH31–HaSABATH33). This clade contained a JMT/carlactonoic acid methyltransferase (CLAMT) subclade and a SAMT subclade. The JMT/CLAMT subclade included AtJMT (AT1G19640), AtCLAMT (AT4G36470), a poplar JMT, rice JMT homologs and four HaSABATH proteins (HaSABATH1, HaSABATH2, HaSABATH31 and HaSABATH33). The SAMT subclade included functionally validated proteins such as CbSAMT and AmSAMT and eight HaSABATH proteins (HaSABATH11–HaSABATH17 and HaSABATH32).

Clade II, the BAMT/BSMT-related clade, contained 51 sequences and was the largest sunflower SABATH clade, with 19 HaSABATH members (HaSABATH3–HaSABATH9, HaSABATH18–HaSABATH21 and HaSABATH23–HaSABATH30). This clade included a Core BSMT subclade and a xanthosine methyltransferase/dimethylxanthine methyltransferase (XMT/DXMT) subclade. All 19 HaSABATH proteins in Clade II were placed in the Core BSMT subclade and formed a sunflower-rich sister group within the sampled tree. The XMT/DXMT subclade contained coffee xanthine methyltransferases and soybean or cotton homologs annotated as XMT/7-methylxanthine methyltransferase (MXMT), but no HaSABATH member.

Other lineages contained 50 sequences, including the outgroup WP_009976433, and were outside Clade I and Clade II. This group included indole-3-acetic acid methyltransferases (IAMTs), gibberellin and farnesoic acid methyltransferases, several monocot aromatic methyltransferases, loganic acid methyltransferase and functionally uncharacterized members. Only HaSABATH10 and HaSABATH22 were assigned to this group. HaSABATH22 clustered with AtIAMT1 and OsIAMT1 and had 68.4% protein identity to AtIAMT1 (E-value = 0), supporting its annotation as a sunflower IAMT-like candidate. HaSABATH10 was positioned near the base of Other lineages, and its function remains to be tested.

BLASTP best hits and phylogenetic assignments were not identical. Although 26 HaSABATH proteins had *Arabidopsis* best hits from the BAMT/BSMT-related lineage, the phylogenetic tree assigned only 19 of them to Clade II, while the remaining seven were mainly located in the SAMT/JMT-related region of Clade I.

### 2.3. Conserved Motifs and Gene Structures

**Figure 2:**
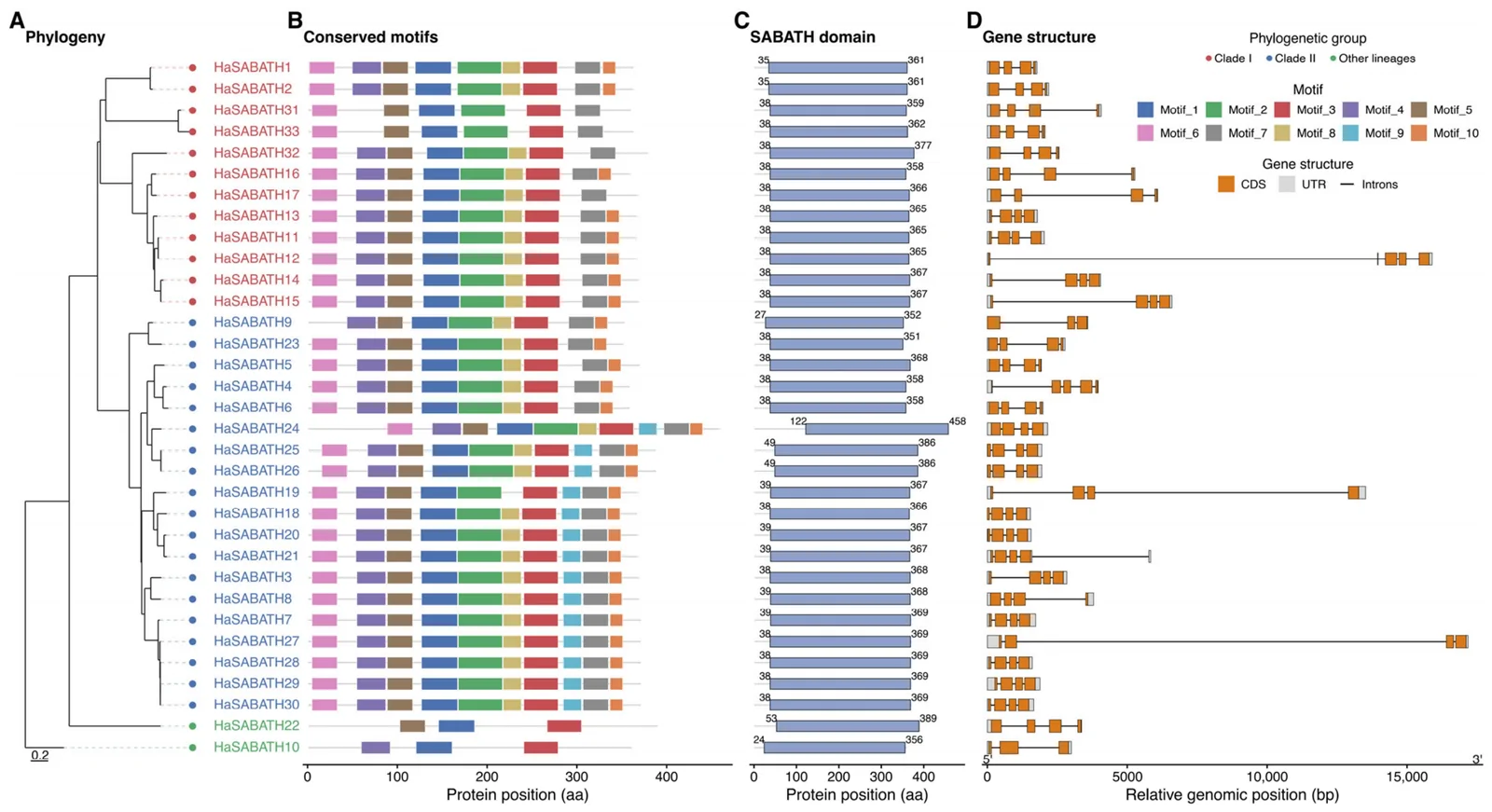
**Conserved motifs, PF03492 domains and gene structures.** (**A**) Intraspecific maximum-likelihood tree of the 33 *HaSABATH* genes with group annotations; (**B**) MEME motif distribution; (**C**) PF03492 domain architecture; (**D**) exon–intron structures. Orange boxes indicate CDS regions, gray boxes indicate untranslated regions (UTRs) and black lines indicate introns.

MEME analysis identified 10 conserved motifs in the 33 HaSABATH proteins (Figure 2 and Figure S2; Table S3a–c). Motif 1 and Motif 3 were present in all 33 members. Motif 2 was present in 31 members and was absent only from the two proteins in Other lineages, HaSABATH10 and HaSABATH22. Consensus sequences, motif occurrences and position weight matrices are provided in Table S3a, S3b and S3c, respectively.

Motif presence differed among phylogenetic groups. Motif 4 was detected in all 19 Clade II members, 10 of 12 Clade I members and one of two members in Other lineages. Motif 8 was detected in 18 of 19 Clade II members and 10 of 12 Clade I members, but in neither member in Other lineages. These motif differences represent descriptive sequence features that distinguish lineages.

Gene-structure analysis showed that 28 of the 33 *HaSABATH* genes (84.8%) contained four gene exons, consistent with exon conservation in the *Arabidopsis* SABATH family [7,23]. *HaSABATH4*, *HaSABATH12* and *HaSABATH21* contained five gene exons, while *HaSABATH9* and *HaSABATH10* contained three. One exon in *HaSABATH21* was confined to an untranslated region (UTR), resulting in four CDS exons. Together, the motif and exon patterns indicate that the SABATH core architecture is conserved while several lineage-associated differences remain detectable.

### 2.4. Chromosomal Localization, Duplication Types and Synteny

**Figure 3:**
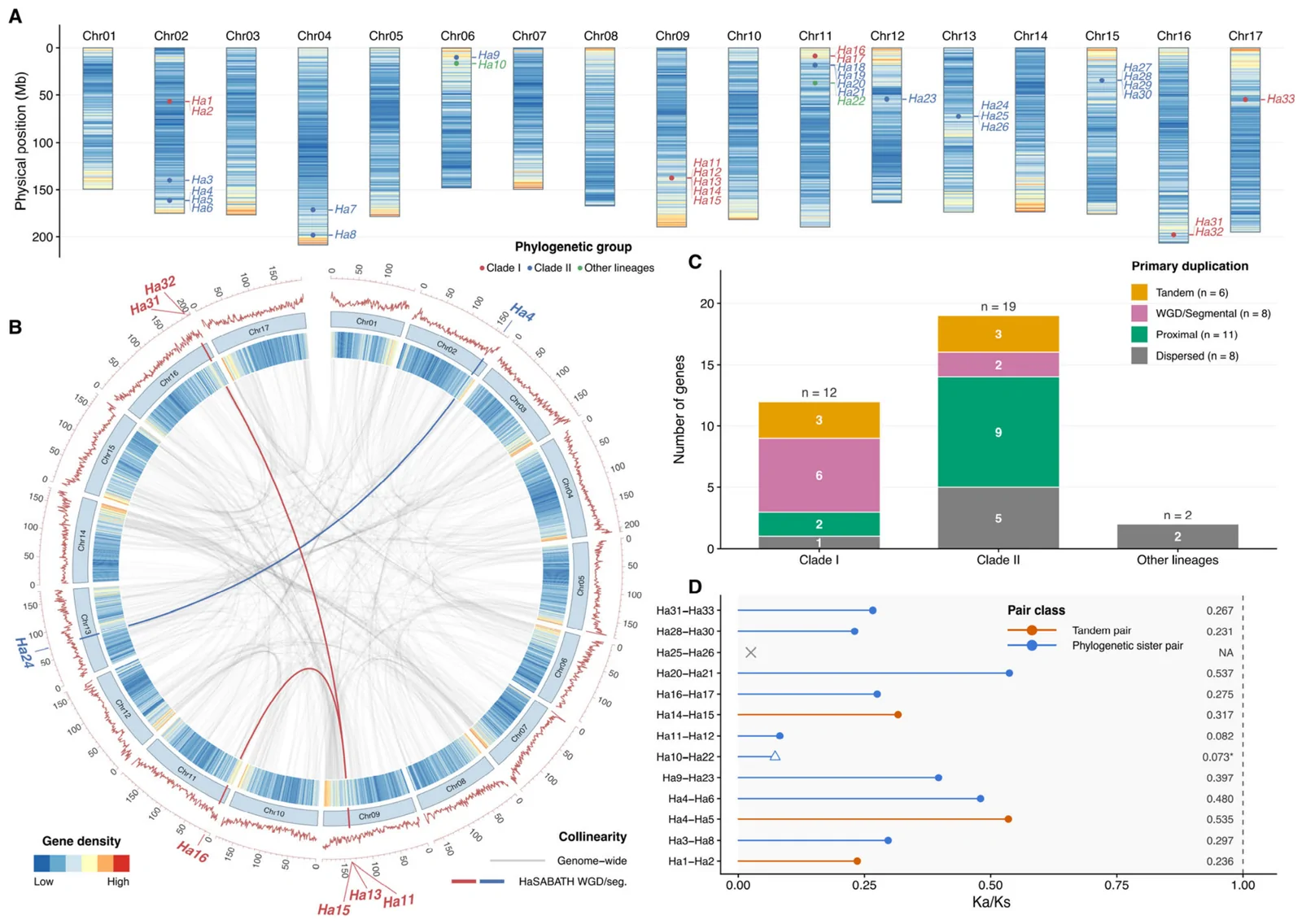
**Chromosomal distribution, duplication patterns and Ka/Ks estimates of *HaSABATH* genes.** (**A**) Physical distribution of the 33 *HaSABATH* genes across 10 of the 17 sunflower chromosomes, with chromosome lengths proportional to physical size; (**B**) genome-wide collinearity and *HaSABATH*-associated WGD/segmental duplication links; (**C**) composition of primary duplication types within each phylogenetic group; (**D**) Ka/Ks estimates for 13 candidate comparisons. In (**D**), circles denote reliable estimates. Ka/Ks could not be estimated for *HaSABATH25–HaSABATH26* because their CDS sequences were identical (gray cross, NA); *HaSABATH10–HaSABATH22* is marked as affected by synonymous-site saturation (open triangle and asterisk). *HaSABATH4* and *HaSABATH15* are classified as WGD/segmental but also retain tandem evidence.

The 33 *HaSABATH* genes were unevenly distributed across 10 chromosomes (Figure 3; Table S2). chr11 contained the largest number of members (seven; *HaSABATH16–HaSABATH22*), followed by chr2 (six; *HaSABATH1–HaSABATH6*). MCScanX identified four tandem duplicate pairs from the sunflower whole-proteome self-BLASTP analysis: *HaSABATH1–HaSABATH2* on chr2 (121.0 kb intergenic gap), *HaSABATH4–HaSABATH5* on chr2 (82.9 kb intergenic gap), *HaSABATH14–HaSABATH15* on chr9 (64.1 kb intergenic gap) and *HaSABATH25–HaSABATH26* on chr13 (6.7 kb intergenic gap; identical CDS sequences). The two members of each tandem pair belonged to the same phylogenetic group. *HaSABATH1–HaSABATH2* and *HaSABATH14–HaSABATH15* belonged to Clade I, whereas *HaSABATH4–HaSABATH5* and *HaSABATH25–HaSABATH26* belonged to Clade II.

Using the nonredundant primary duplication category, eight genes were assigned to the whole-genome duplication (WGD)/segmental category and formed three cross-chromosomal blocks: *HaSABATH4* (chr2) with *HaSABATH24* (chr13), *HaSABATH11* (chr9) with *HaSABATH16* (chr11), and *HaSABATH13* and *HaSABATH15* (chr9) with *HaSABATH31* and *HaSABATH32* (chr16). Eleven genes were classified as proximal, mainly on chr11 (*HaSABATH17–HaSABATH21*) and chr15 (*HaSABATH27–HaSABATH30*), with *HaSABATH6* on chr2 and *HaSABATH12* on chr9. Eight genes were classified as dispersed (*HaSABATH3*, *HaSABATH7*, *HaSABATH8*, *HaSABATH9*, *HaSABATH10*, *HaSABATH22*, *HaSABATH23* and *HaSABATH33*), and six genes were classified as tandem in the primary category. No singleton *HaSABATH* gene was detected. *HaSABATH4* and *HaSABATH15* were counted as WGD/segmental in the primary classification but also had local tandem evidence with *HaSABATH5* and *HaSABATH14*, respectively; all overlapping evidence is retained in Table S2. These results suggest that local tandem duplication, proximal duplication and chromosomal segmental duplication jointly contributed to *HaSABATH* family expansion.

**Figure 4:**
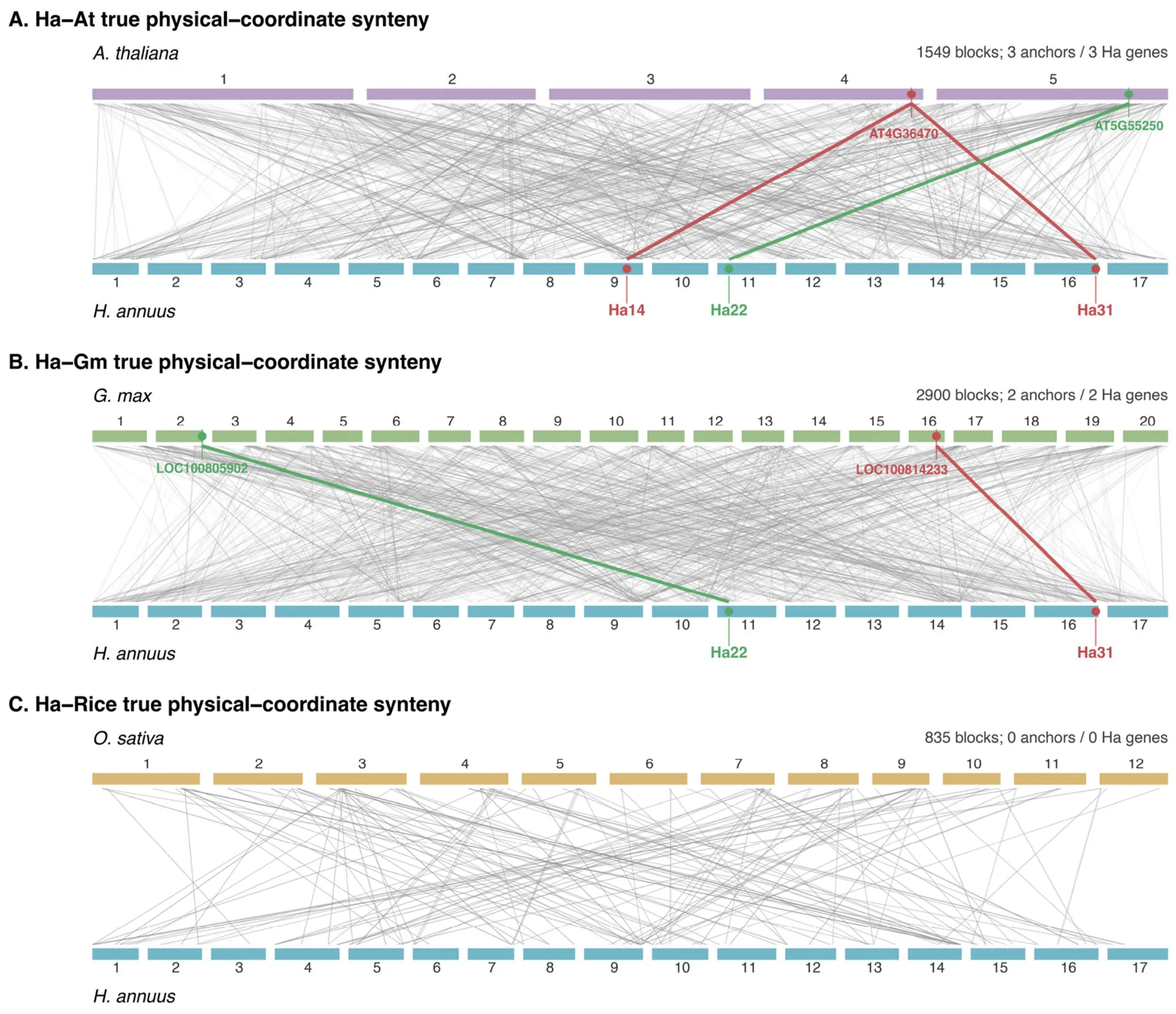
**Cross-species synteny between sunflower and *Arabidopsis*, soybean and rice.** (**A**) Sunflower–*Arabidopsis* (Ha-At); (**B**) sunflower–soybean (Ha-Gm); (**C**) sunflower–rice (Ha-Rice). Each panel is based on physical chromosome coordinates and independent two-species BLASTP/MCScanX analyses. Gray lines show mappable cross-species anchors in the background; colored links and labels identify direct anchors involving *HaSABATH* genes and their partner genes. No direct *HaSABATH* anchor was detected in Ha-Rice. Numbers along the chromosome bars denote chromosome identifiers. Each panel also reports total collinearity blocks, direct *HaSABATH*-associated anchor pairs and distinct *HaSABATH* genes.

Cross-species MCScanX analysis detected a small number of direct *HaSABATH* syntenic anchors (Figure 4; Table 2). In the Ha-At comparison, direct anchors involved *HaSABATH14* with *AT4G36470*, *HaSABATH22* with *AT5G55250* and *HaSABATH31* with *AT4G36470*. In the Ha-Gm comparison, direct anchors involved *HaSABATH22* with *LOC100805902* and *HaSABATH31* with *LOC100814233*. No direct *HaSABATH* anchor was detected in the Ha-Rice comparison. These results indicate that direct cross-species *HaSABATH* anchors are limited under the current MCScanX parameter settings and are mainly retained in dicot comparisons.

### 2.5. Ka/Ks Selection Pressure

Thirteen candidate comparisons were retained for Ka/Ks analysis (Figure 3D; Table S4). Eleven comparisons yielded reliable estimates, all below 1. The reliable *ω* range was 0.082–0.537. *HaSABATH11–HaSABATH12* had the lowest value (0.082), and *HaSABATH20–HaSABATH21* had one of the highest values (0.537). Ka/Ks could not be estimated for *HaSABATH25*–*HaSABATH26* because their CDS sequences were identical. *HaSABATH10–HaSABATH22* yielded an exceptionally high Li–Pamilo–Bianchi 1993 (LPB93) *d*_S_ estimate (9.593), consistent with extensive synonymous-site saturation and making its *ω* estimate unreliable for biological interpretation. The estimate was retained in Table S4 for transparency but excluded from the summary of 11 reliable *ω* values.

The three reliable tandem or primary duplicate comparisons had a mean *ω* of 0.362, while the eight reliable exploratory sister-pair comparisons had a mean *ω* of 0.321. These comparable ω estimates suggest that the analyzed recent duplicate and sister pairs were mainly under purifying constraint rather than positive selection.

### 2.6. Promoter Cis-Element Analysis

**Figure 5:**
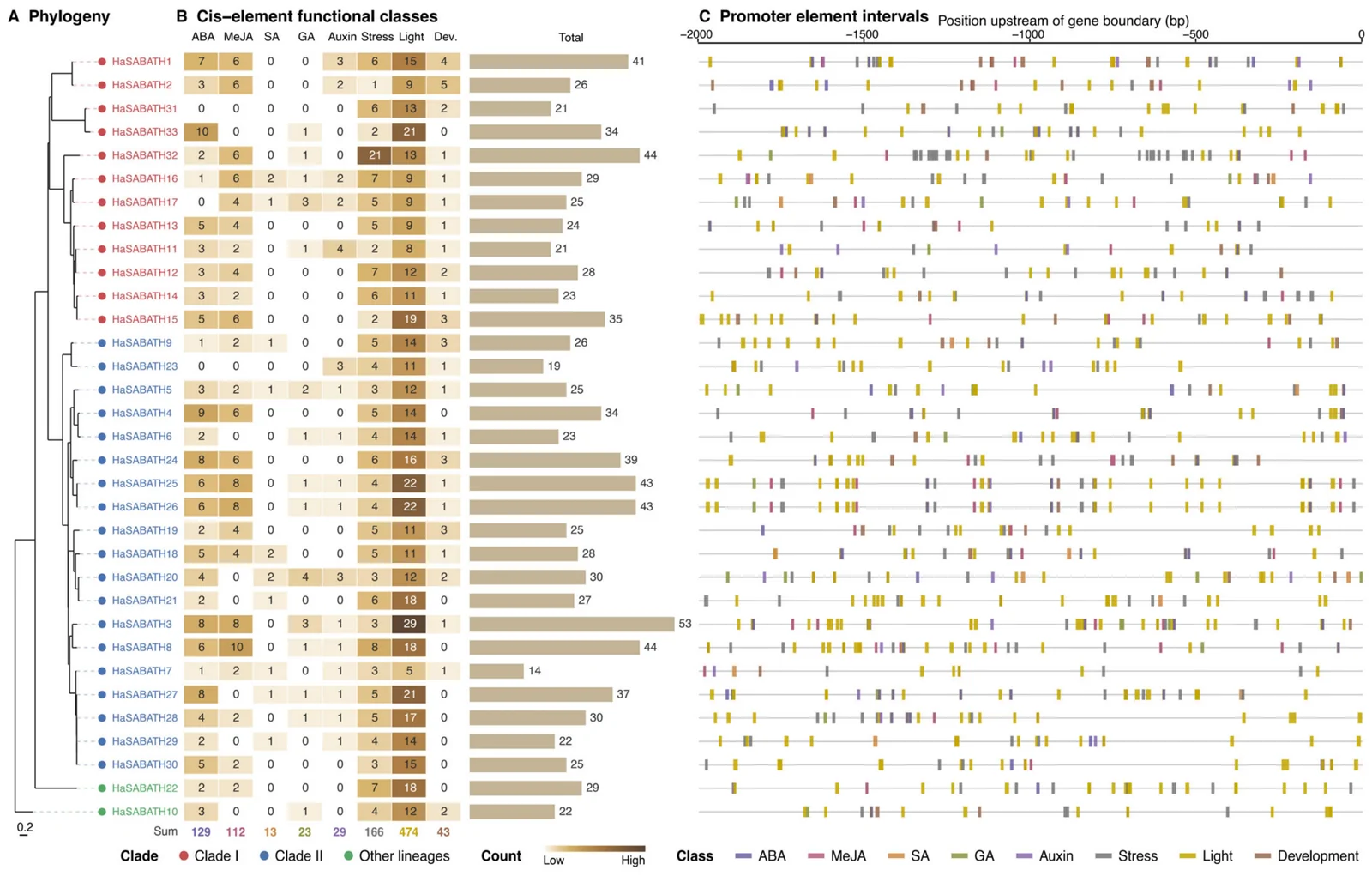
**Promoter cis-element distribution in *HaSABATH* genes.** (**A**) Intraspecific phylogeny and group annotation; (**B**) per-gene counts of eight curated functional cis-regulatory element categories, with horizontal bars on the right showing the sum of category counts for each gene (Total); (**C**) element positions within strand-aware 2 kb regions upstream of annotated gene boundaries. The categories are abscisic acid (ABA), methyl jasmonate (MeJA), salicylic acid (SA), gibberellin (GA), Auxin, Stress, Light and Development. Record-level annotations and category statistics are provided in Table S5a and S5b, respectively.

Promoter analysis predicted light-, stress- and hormone-related cis-regulatory elements across *HaSABATH* promoters (Figure 5; Table S5a,b). Light-response elements were the most abundant category, followed by Stress, abscisic acid (ABA) and methyl jasmonate (MeJA) elements. Mann–Whitney *U* tests comparing Clade II genes with other genes did not detect a significant difference after false discovery rate (FDR) correction for any of the eight functional categories.

### 2.7. Multidimensional Transcriptome Expression Profiles

**Figure 6:**
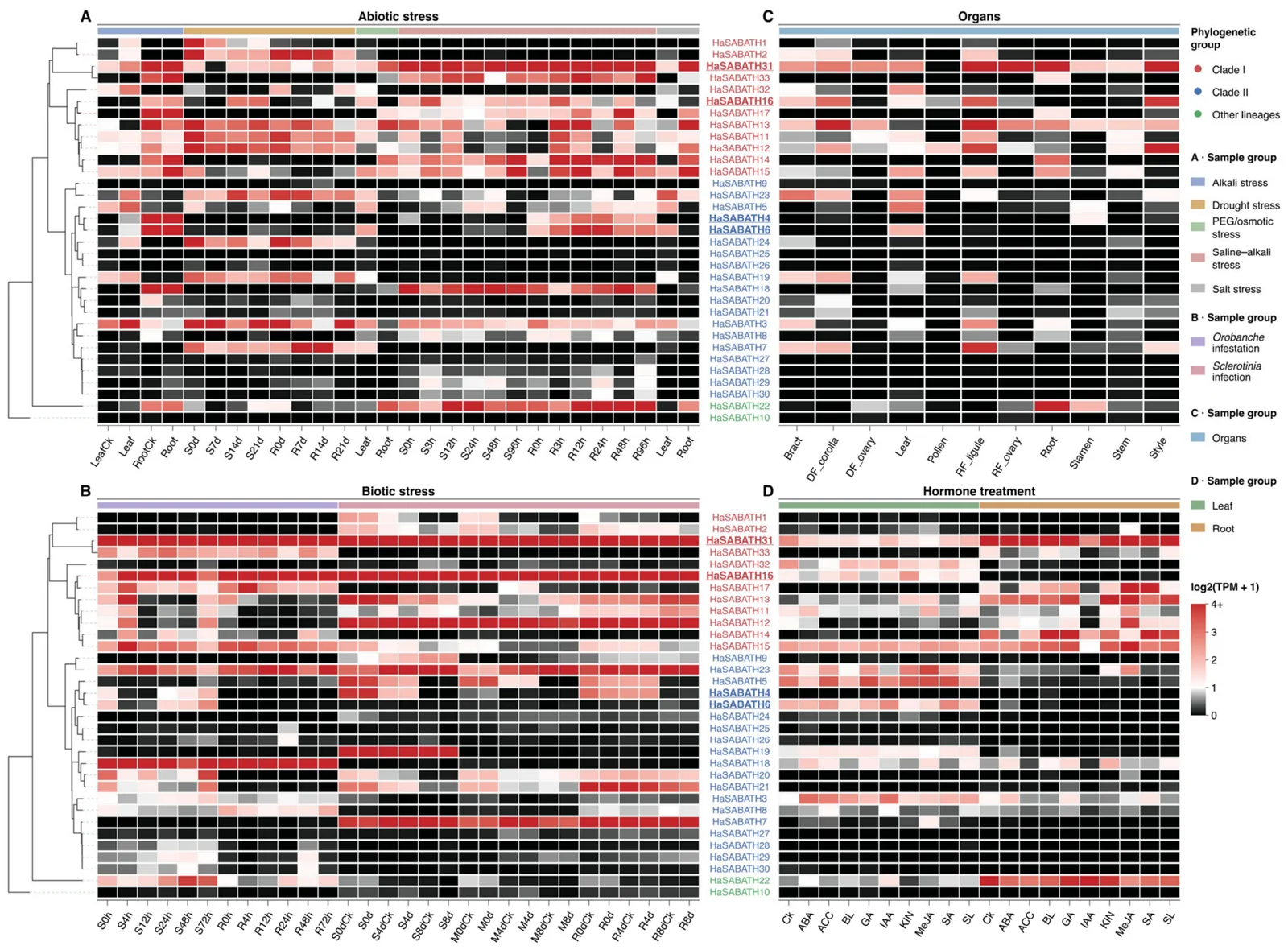
**Multidimensional transcriptome expression profiles of *HaSABATH* genes.** Expression values are log_2_(mean TPM + 1) values without row scaling; values above 4 are displayed at the upper limit of the color scale (4+). Red, blue and green gene names indicate Clade I, Clade II and Other lineages, respectively; *HaSABATH4*, *HaSABATH6*, *HaSABATH16* and *HaSABATH31* are marked in bold and underlined. The top color bars indicate the sample groups in each panel. (**A**) Abiotic stress. The panel includes alkaline stress, drought, polyethylene glycol (PEG) osmotic stress, combined saline–alkali stress and salt stress. S and R in the drought data denote the sensitive material K55 (DS) and the drought-tolerant material K58 (DT), respectively; S and R in the combined saline–alkali data denote the sensitive material K-7 and the tolerant material K-27, respectively. Leaf and Root denote leaf and root tissues, respectively. (**B**) Biotic stress. The panel includes broomrape infestation and *Sclerotinia sclerotiorum* infection. S and R in the broomrape data denote susceptible and resistant materials, respectively; S, M and R in the *Sclerotinia* data correspond to HA89 (susceptible), HA853 (moderately tolerant) and RK416 (more tolerant), respectively. (**C**) Organs. Bract, Leaf, Pollen, Root, Stamen and Stem denote bracts, leaves, pollen, roots, stamens and stems, respectively. DF and RF denote disc and ray florets, respectively; DF_corolla and DF_ovary denote disc floret corollas and ovaries, respectively, and RF_ligule and RF_ovary denote ray floret corollas and ovaries, respectively. Style denotes pooled stigma and style samples. (**D**) Hormone treatments. The panel shows expression profiles under hormone treatments in leaves and roots. ABA, ACC, BL, GA, IAA, KIN, MeJA, SA and SL denote abscisic acid, 1-aminocyclopropane-1-carboxylic acid, brassinosteroid, gibberellin, indole-3-acetic acid, kinetin, methyl jasmonate, salicylic acid and strigolactone treatments, respectively. Ck denotes the control for the corresponding experiment; h and d denote hours and days, respectively; TPM denotes transcripts per million. Details of materials, treatments, sampling times, replicates and libraries are provided in Table S6a, and mean TPM values are provided in Table S6c.

The descriptive expression atlas constructed from public RNA sequencing (RNA-seq) data revealed substantial expression heterogeneity across the 33 genes and 87 conditions (Figure 6; Table S6a,c). *HaSABATH16* had the highest peak expression value (log_2_(TPM + 1) = 9.04, where TPM denotes transcripts per million), followed by *HaSABATH31*, *HaSABATH23*, *HaSABATH12*, *HaSABATH14* and *HaSABATH13*, with maxima of 6.86–7.39. *HaSABATH31* showed the broadest expression, with log_2_(TPM + 1) > 3 in 58 of 87 conditions. By contrast, *HaSABATH1*, *HaSABATH2*, *HaSABATH9*, *HaSABATH10*, *HaSABATH24* and *HaSABATH27–HaSABATH30* were weakly expressed or nearly silent in most conditions. *HaSABATH25* and *HaSABATH26* have identical annotated exon sequences, and their member-specific expression estimates are limited by ambiguity in read assignment. The descriptive grand mean of log_2_(TPM + 1) was 1.60 for Clade I and 0.68 for Clade II. Within Clade I, the corresponding means for WGD/segmental and non-WGD members were 2.15 and 1.04, respectively. Comparisons across study sources and summary methods, expression-breadth thresholds, and member or source omissions are provided in Tables S8–S12.

Abiotic stress datasets revealed different candidate members across combined saline–alkali stress, salt stress, alkaline stress, drought and polyethylene glycol (PEG) osmotic stress. The clearest genotype-associated pattern involved *HaSABATH4* and *HaSABATH6*. Following combined saline–alkali treatment, *HaSABATH4* expression in K-27 roots increased significantly at 12 and 24 h relative to its 0 h level, whereas *HaSABATH6* expression increased significantly at all subsequent sampling times relative to its 0 h level; their peak mean TPM values were 8.33 and 14.65, respectively. By contrast, the two genes were undetectable or expressed at very low levels after treatment in K-7, with mean TPM ranges of 0–0.10 and 0–0.32, respectively (Figure 6A; Table S6c,e). *HaSABATH14* reached log_2_(TPM + 1) = 6.90 at 48 h in K-27, but it also reached 5.75 at 96 h in K-7 and is better interpreted as a responsive member rather than a member specific to the tolerant genotype. *HaSABATH22* was expressed in the combined saline–alkali stress root panel and in indole-3-acetic acid (IAA) and salicylic acid (SA) root hormone treatments, which is consistent with its IAMT-like candidate annotation. Under salt stress in roots, *HaSABATH13* had the highest expression, reaching log_2_(TPM + 1) = 6.86. *HaSABATH31*, *HaSABATH15* and *HaSABATH14* were also more highly expressed in roots than in leaves. Under alkaline stress in roots, *HaSABATH17*, *HaSABATH14* and *HaSABATH18* all had log_2_(TPM + 1) values above 5. In the drought panel, *HaSABATH3* showed higher expression in the drought-tolerant material at 21 d, whereas *HaSABATH12* and *HaSABATH13* peaked in the sensitive material at 0 d. In the PEG panel, *HaSABATH13*, *HaSABATH22*, *HaSABATH31* and *HaSABATH14* showed relatively high expression in root samples, whereas *HaSABATH15* and *HaSABATH3* showed relatively high expression in leaf samples. Overall, abiotic stress expression was not dominated by a single clade or duplication type.

The biotic stress panels supported different interpretations of the expression patterns associated with *Sclerotinia* infection and broomrape infestation. *HaSABATH16* had the highest expression value in the whole matrix and reached log_2_(TPM + 1) = 9.04 in HA89 at 8 days post inoculation (dpi) and 8.93 in HA853 at 8 dpi. The corresponding mock controls at similar time points also showed high expression, including 8.90 in HA89 and 8.53 in HA853. *HaSABATH16* is therefore best described as a capitulum-associated candidate with high expression rather than a gene specifically induced by the pathogen. *HaSABATH12*, *HaSABATH23* and *HaSABATH31* also showed high expression or some differences between inoculated and mock-inoculated samples in the *Sclerotinia* panel. In the broomrape panel, *HaSABATH31* had a mean log_2_(TPM + 1) value approximately 1.41 higher in resistant samples than in susceptible samples. *HaSABATH23* and *HaSABATH16* also tended to show higher expression in the resistant genotype, whereas *HaSABATH20*, *HaSABATH21* and *HaSABATH22* showed higher expression in the susceptible genotype.

Hormone and organ panels further distinguished the expression contexts of candidate members. Treatment abbreviations denote 1-aminocyclopropane-1-carboxylic acid (ACC), brassinosteroids (BL), gibberellin (GA), kinetin (KIN) and strigolactone (SL). *HaSABATH13* and *HaSABATH15* reached log_2_(TPM + 1) values of 6.59 and 6.03 in MeJA-treated roots, respectively, suggesting a MeJA-related expression context. *HaSABATH31* retained high expression in roots treated with ABA, ACC, BL, GA, KIN, MeJA and SL, consistent with broad expression. In organ samples, *HaSABATH13* and *HaSABATH31* reached 5.65 and 5.22 in ray floret corollas, respectively, and *HaSABATH12* reached 4.98 in pooled stigma and style samples. Collectively, *HaSABATH31* represents a broadly expressed candidate for follow-up; *HaSABATH4* and *HaSABATH6* are combined saline–alkali stress candidates; *HaSABATH16*, *HaSABATH23* and *HaSABATH12* are candidates from the capitulum or biotic stress panels; and *HaSABATH13* and *HaSABATH15* show higher expression in MeJA-treated or salt-stressed root samples.

### 2.8. Protein-Structure-Prediction Confidence

**Figure 7:**
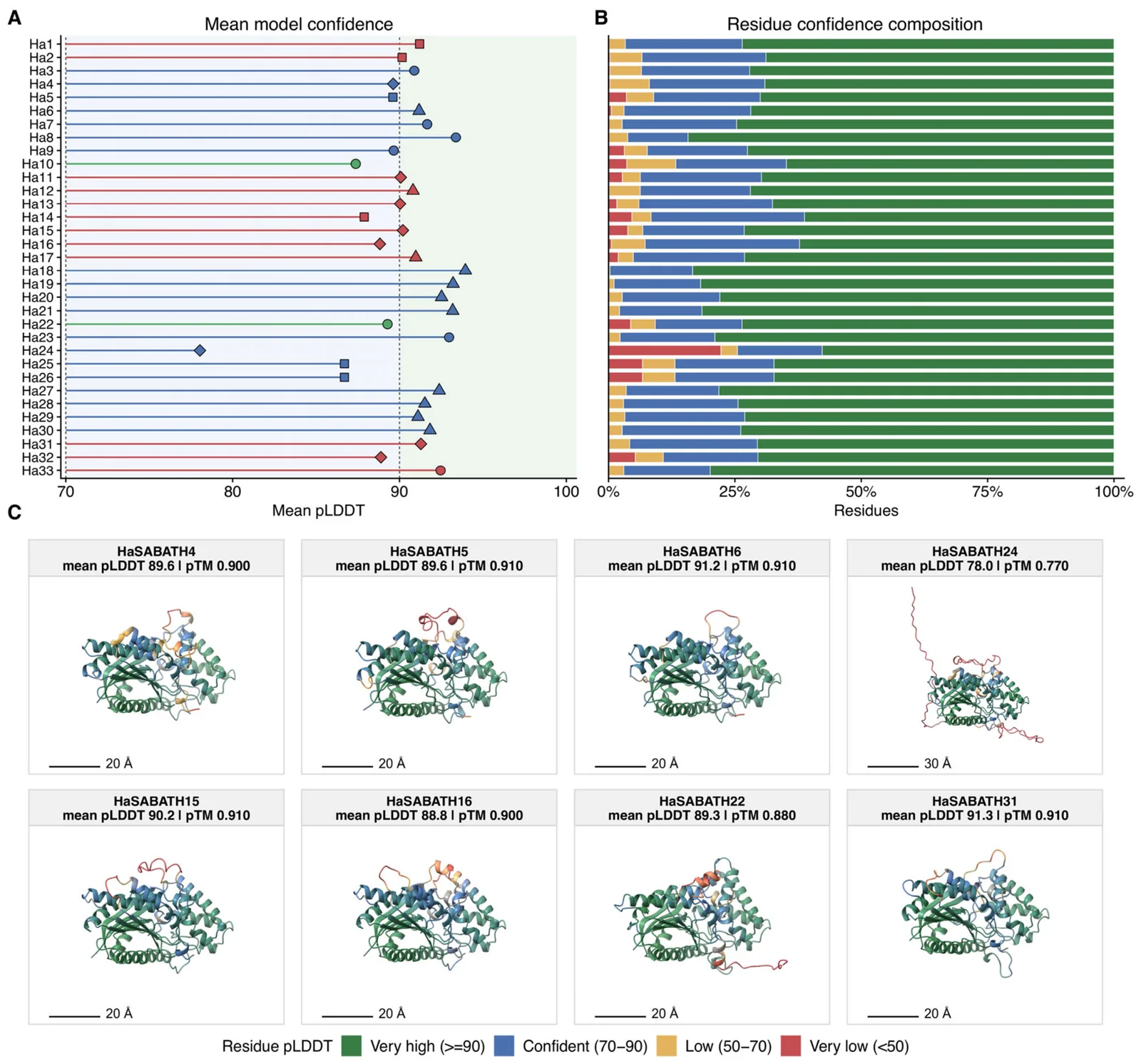
**Confidence assessment and representative predicted structures of HaSABATH proteins.** Panels show mean pLDDT for 33 rank-1 models (**A**), residue-level pLDDT composition (**B**), and pLDDT-colored ChimeraX ribbon views of eight representative proteins (**C**). Mean pLDDT and pTM values are labeled. In panel (**C**), the scale bar is 30 Å for HaSABATH24 and 20 Å for the other seven structures. In panel (**A**), red, blue and green indicate Clade I, Clade II and Other lineages, respectively. Squares, diamonds, triangles and circles indicate the primary duplication classes Tandem, WGD/Segmental, Proximal and Dispersed, respectively. The models illustrate prediction confidence and overall architecture and are not used to assign substrate specificity or enzyme activity. pLDDT, predicted local distance difference test; pTM, predicted template modeling score.

AlphaFold2 prediction was performed for all 33 HaSABATH proteins to evaluate model confidence (Figure 7). The full structure-prediction confidence metrics are provided in Table S7. The median mean predicted local distance difference test (pLDDT) value was 90.89, with a range of 78.04–93.95. Twenty-two models had mean pLDDT ≥ 90. The average predicted template modeling score (pTM) was 0.902, with a range of 0.77–0.93. These values indicate generally high model confidence for most HaSABATH proteins, while terminal or loop regions varied in local confidence. HaSABATH24 was the only member with mean pLDDT below 80 (78.04; pTM = 0.77), and its low-confidence residues indicated uncertainty in the corresponding local structural predictions. These confidence metrics assess the predicted models and do not establish substrate specificity, catalytic activity or biological function.

Eight representative proteins were visualized as individual ChimeraX ribbon structures. The set included four Clade II proteins: the WGD/segmental bridge HaSABATH4, its tandem partner HaSABATH5, the proximal duplicate and saline–alkali stress candidate HaSABATH6 and the segmental partner HaSABATH24. It also included the Clade I WGD-tandem bridge HaSABATH15, the highly expressed capitulum-associated candidate HaSABATH16, the IAMT-like candidate HaSABATH22 from Other lineages and the broadly expressed WGD/segmental member HaSABATH31. The eight representatives had mean pLDDT values of 78.04–91.28 and pTM values of 0.77–0.91. Visual inspection of the individual predicted models suggested a broadly similar overall SABATH-like architecture; no quantitative structural superposition was used to infer functional equivalence. Model confidence and visual similarity do not establish substrate-pocket differences, enzyme activity or biological function.

## 3. Discussion

### 3.1. Sunflower Members Are Concentrated in the BAMT/BSMT-Related Clade

BLASTP best hits indicated that 26 of the 33 HaSABATH proteins were closest to *Arabidopsis* reference proteins in the BAMT/BSMT-related lineage, whereas the multispecies tree placed 19 HaSABATH proteins in the Clade II Core BSMT subclade. Within the present sampling framework, sunflower had more Clade II members than *Arabidopsis*, soybean, cotton or rice in the corresponding clade.

The functions of characterized reference enzymes provide clues for testing whether sunflower Clade II members participate in floral volatile or defense-related metabolism. Methyl benzoate and methyl salicylate produced by BAMT/BSMT enzymes are important floral volatiles and defense-related signals [9,16,17,18,31]. Because floral volatile compounds can contribute to pollinator attraction [18], this lineage warrants future study in the context of sunflower floral volatile metabolism. Experimental work in *Populus* has shown that a single amino-acid substitution can switch substrate preference in a SABATH enzyme [20]. Thus, within the present sampling framework, the expanded set of sunflower Clade II genes provides candidates for studying expression divergence and potential functional differences related to aromatic carboxylic acid methylation, but substrate specificity cannot be inferred from copy number alone. These 19 Clade II members require enzymatic validation, combined with metabolite analysis to test their potential links to floral volatiles or defense.

The selection and synteny analyses further define the scope of the evolutionary interpretation. The 13 Ka/Ks comparisons involved preselected duplicate or phylogenetic sister pairs and did not constitute an exhaustive family-wide scan; the 11 reliable *ω* estimates below 1 support purifying constraint only for those analyzed comparisons. Likewise, the three, two and zero direct *HaSABATH* anchors detected in the *Arabidopsis*, soybean and rice comparisons, respectively, depend on the reference sets, sequence divergence and current BLASTP/MCScanX parameters. They should not be interpreted as direct evidence of lineage-specific gene loss or as a quantitative ranking of synteny among species.

### 3.2. Expression Divergence and Candidate Genes in Stress Responses

The transcriptome analysis revealed both between-clade and within-clade expression differences. Across the sampled conditions, Clade I members generally showed higher expression levels and broader expression, while many Clade II members were weakly expressed or condition-biased (Figure 6; Table S6a,c). Within Clade I, the WGD/segmental duplication group had higher mean expression and greater expression breadth than the non-WGD group. Among all members, *HaSABATH31* had the broadest expression in the datasets compiled in this study and is better viewed as a candidate expressed in multiple contexts than as a gene specific to a single stress. For the more numerous Clade II members, expression biases suggest tissues or treatment contexts to prioritize when investigating their metabolic roles. The *Populus* SABATH family shows divergence in both expression patterns and catalytic properties [20]. A key question linking expression divergence to metabolic function is therefore whether related sunflower members retain similar catalytic functions but are expressed in different contexts or whether their expression biases are accompanied by changes in substrate preference.

No statistically significant differences between Clade II genes and other genes were detected for any of the eight functional cis-element categories after FDR correction. These category counts provided no direct evidence explaining the observed expression divergence, and the presence of short predicted motifs does not establish transcription-factor binding. Previous SABATH studies have linked expression changes to specific upstream regulation and metabolic consequences. For example, regulatory changes in the *Arabidopsis* miR163 system can affect the expression of specific SABATH targets and the metabolite profile [32]. In wheat, nuclear TaBZR1 recruits the histone acetyltransferase TaHAG1, increasing H3 acetylation and transcription at the *TaSAMT1-B* locus. TaSAMT1-B is predominantly cytoplasmic and catalyzes the formation of methyl salicylate (MeSA) from SA, contributing to the regulation of freezing tolerance in wheat [33]. These functional studies provide regulatory explanations beyond predicted element-category counts, but the specific sources of regulation of HaSABATH members have not been identified in this study.

Among the abiotic stress datasets, *HaSABATH4* and *HaSABATH6* are priority candidates for root responses to combined saline–alkali stress. *HaSABATH4* expression increased mainly at 12–24 h in K-27, whereas *HaSABATH6* already had higher baseline expression in K-27 than in K-7 and increased further after treatment; expression of both genes remained extremely low overall in K-7. The source study reported sustained upregulation of the BAMT locus *LOC110899290* in K-27 and supported its expression trend by qRT-PCR [6]; this locus corresponds to *HaSABATH4* in this study. *HaSABATH4* and *HaSABATH6* have different duplication backgrounds. *HaSABATH4* has both chr2-chr13 WGD/segmental syntenic evidence and local tandem-duplication evidence with *HaSABATH5*, whereas *HaSABATH6* is a nearby proximal duplicate and a strongly supported sister of *HaSABATH4*. The different temporal patterns of these related members may reflect differential responses to upstream signals. Even if their catalytic functions are similar, differences in expression timing may allow them to participate in small-molecule conversion at different stages.

*HaSABATH13* and *HaSABATH15* showed high expression in MeJA-treated roots. Expression of *Arabidopsis AtBSMT1*, whose encoded enzyme uses SA and benzoic acid (BA) as substrates, can be induced by MeJA [17], and the rice jasmonic acid (JA)-responsive transcription factor MYC2 can directly bind and activate the *BSMT1* promoter [34]. The MeJA-associated expression of *HaSABATH13* and *HaSABATH15* may therefore involve jasmonate-mediated transcriptional regulation of members related to aromatic carboxylic acid methylation. In addition, *HaSABATH13* showed high expression in salt-stressed roots and at early time points of combined saline–alkali stress in K-27, indicating that its high-expression contexts were not restricted to MeJA treatment.

*HaSABATH16* had the highest expression value in the whole matrix and was especially highly expressed in capitulum samples. Because mock controls in the same *Sclerotinia* dataset also showed high expression at similar time points, *HaSABATH16* is best interpreted as a capitulum-associated candidate with high expression rather than a gene specifically induced by the pathogen. *HaSABATH16* belongs to the Clade I SAMT subclade and to the chr9-chr11 WGD/segmental block. In petunia flowers, PhbZIP3 can bind and activate the *PhBSMT* promoter [35], showing that organ- and development-associated transcriptional programs can regulate this type of gene. Together with the characterized roles of floral SAMTs in MeSA formation [8], these observations support investigating *HaSABATH16* in floral aromatic carboxylic acid metabolism. Any potential pathogen-associated role should be distinguished from the organ-expression background.

The broomrape dataset showed higher mean expression of *HaSABATH31*, *HaSABATH16*, *HaSABATH18* and *HaSABATH23* in the resistant genotype, whereas *HaSABATH20*, *HaSABATH22* and *HaSABATH21* had higher mean expression in the susceptible genotype. These biases were already present at 0 h, indicating that the pretreatment expression background is an important starting point for interpreting subsequent differences. The differences may be associated with metabolic states that influence resistance or may simply be correlates of the genotypic background; the single resistant–susceptible genotype pair examined here cannot distinguish these explanations. Characterized SABATH members can participate in stress responses or developmental regulation through small-molecule methylation [14,33], so the functional significance of higher expression depends on the actual substrate and the consequences of its methylation. Further work needs to determine whether these expression biases remain consistently associated with resistance in other resistant and susceptible genotypes and whether candidate members affect parasitism by altering the balance between the relevant substrates and methyl esters. This would help distinguish metabolic processes affecting resistance from associations specific to a particular genotypic background.

### 3.3. Limitations and Perspectives

First, phylogenetic classification was based on a 148-sequence ML tree, and some nodes had moderate ultrafast bootstrap (UFBoot) support. Additional taxon sampling and more complex evolutionary models may further improve topology stability. Second, substrate specificity and enzyme activity were not experimentally tested; putative functional contexts were assigned tentatively from sequence similarity and phylogenetic position, while predicted structures were used only to assess model confidence and overall architecture. Because the present analysis focused on monomeric model confidence rather than protein–ligand complexes, ColabFold/AlphaFold2 was used only for confidence screening and not to infer substrate binding or enzyme activity. Finally, expression analysis was based on multiple public RNA sequencing (RNA-seq) projects. Cross-project averages were used for pattern description and candidate screening, and priority candidate genes require independent experimental validation.

Future work should test all 19 Clade II members in biochemical substrate assays to determine whether sunflower contains enzymes with BAMT, BSMT or other aromatic carboxylic acid methyltransferase activities. *HaSABATH6* can be prioritized for qRT-PCR expression validation under combined saline–alkali stress and *HaSABATH4* and *HaSABATH6* for promoter activity assays, gene editing or transient expression studies. Broadly expressed members such as *HaSABATH31* and members with high expression in floral organs such as *HaSABATH16* should be evaluated in floral volatile, hormone and defense contexts. Structure-guided mutagenesis of conserved and lineage-variable residues could further link the phylogenetic patterns observed here to biochemical mechanisms.

## 4. Materials and Methods

### 4.1. Genome Data Sources

Genome sequences, the General Feature Format version 3 (GFF3) annotation and protein sequences of the sunflower HanXRQr2.0-SUNRISE reference genome (GCF_002127325.2) were downloaded from the National Center for Biotechnology Information (NCBI). The GFF3 annotation contained 83,308 unique gene or pseudogene identifiers. The gene-level representative protein FASTA comprised 57,237 nonredundant sequences and was used for HMMER, BLASTP and protein-level homology searches. Of the 81,015 unique nonpseudogene gene models, 79,723 LOC-identified gene records were used to construct the MCScanX GFF for protein-based nuclear genome collinearity analysis, consistent with the identifier system used in the homology-search input. The remaining 1292 non-LOC models comprised 1170 tRNA genes, 11 rRNA genes, and 111 organellar protein-coding genes and were outside the scope of this analysis. Physical coordinates of the 17 chromosomes and annotated genes were used for chromosomal localization and MCScanX input construction. Protein sequences for *Arabidopsis thaliana* TAIR10.1, soybean Wm82.a4.v1, upland cotton NBI-AD1_v1.1 and rice IRGSP-1.0 were obtained from NCBI and Phytozome v13. Classification information for the 24 *Arabidopsis* SABATH members was based on published studies [7,21] and the *Arabidopsis* Information Resource (TAIR) [36].

Functionally characterized SABATH reference sequences used for the multispecies phylogeny included GenBank entries from snapdragon (*A. majus*), *Clarkia breweri*, coffee (*Coffea* spp.), maize (*Zea mays*), Madagascar periwinkle (*Catharanthus roseus*) and tomato (*Solanum lycopersicum*). The reference set included representative enzymes and SABATH sequences from primary functional and genome-wide studies [8,9,10,12,13,14,15,16,17,20,22,29]. The SABATH-like protein MaSABATH (WP_009976433) from *Mycobacterium avium* was used as the outgroup. Species information, sequence roles and clade annotations for the 148 phylogenetic sequences are listed in Table S1 in Supplementary Tables S1–S7.

### 4.2. Identification of HaSABATH Genes

The hidden Markov model (HMM) profile of Methyltransf_7 (PF03492.22) was downloaded from Pfam [37]. HMMER v3.4 hmmsearch [38] was run with the --cut_tc option against the sunflower proteome of 57,237 sequences. Candidate members were retained using the following criteria: HMM model-coordinate coverage (the proportion of the model length spanned by the matched HMM coordinates; see the note to Table S2) of at least 70%, and BLASTP (NCBI BLAST+ v2.12.0) [39] verification against 24 *Arabidopsis* SABATH proteins with an E-value threshold of 1 × 10^−5^. Protein lengths were restricted to 276–460 aa, approximately 75–125% of the mean length of the 24 *Arabidopsis* SABATH reference proteins used in this study and consistent with the length-filtering range applied to reference sequences from other species. The retained genes were named *HaSABATH1* to *HaSABATH33* according to chromosomal position. LOC IDs, chromosomal coordinates, protein properties, domain coordinates, phylogenetic groups, phylogeny-based putative SABATH types and duplication classes are listed in Table S2. The phylogeny-based putative SABATH type is provided only as tentative functional context and does not constitute evidence of experimentally verified enzyme activity.

### 4.3. Physicochemical Property Analysis

Biopython v1.87 ProteinAnalysis [40] was used to calculate molecular weight (MW), isoelectric point (pI), grand average of hydropathicity (GRAVY; Kyte–Doolittle method) and instability index (Guruprasad method). The aliphatic index was calculated separately using the Ikai formula [41], AI = *X*(Ala) + 2.9X(Val) + 3.9[*X*(Ile) + *X*(Leu)], where *X* denotes mole percentage.

### 4.4. Phylogenetic Analysis and Clade Classification

Two maximum-likelihood (ML) trees were constructed. The intraspecific tree contained only the 33 HaSABATH sequences and was used for gene-structure and expression-profile ordering. The multispecies tree contained 148 sequences, including 33 HaSABATH proteins and 115 reference sequences from *Arabidopsis*, rice, soybean, cotton and functionally characterized GenBank entries and was used for phylogenetic classification. The complete multispecies tree with full tip labels is provided as Figure S1 in Supplementary Figures S1 and S2. Methods and results for outgroup sensitivity analyses are provided in the Supplementary Robustness Analyses (Table S13).

All sequences were aligned with MAFFT v7.526 using the --auto option [42]. The alignments for both trees were trimmed with trimAl v1.4 using -automated1 [43]. ML trees were inferred using IQ-TREE v2.3.6 [44,45]. ModelFinder selected Q.plant+I+G4 for the 33-sequence intraspecific tree and Q.plant+R5 for the 148-sequence multispecies tree [46]. Branch support was assessed with 1000 ultrafast bootstrap (UFBoot) replicates [47].

### 4.5. Conserved Motif and Gene-Structure Analysis

Conserved protein motifs were identified using the online MEME Suite v5.5.9 [48] with the options -protein -mod anr -nmotifs 10 -minw 6 -maxw 50. Exon–intron composition and exon lengths were extracted from the GFF3 annotation file for gene-structure visualization. The 10 motif models, motif occurrences and position weight matrices are provided in Table S3a, S3b and S3c, respectively; motif logos are provided in Figure S2. Protein, coding sequence (CDS) and promoter FASTA files used in this study are provided in Supplementary Sequence Data.

### 4.6. Chromosomal Localization and Synteny Analysis

Chromosomal physical coordinates were extracted from GFF3 for chromosome mapping. BLAST+ v2.12.0 and MCScanX [49] were used for homology and synteny analyses. The intraspecific analysis used a self-BLASTP search of the sunflower whole proteome of 57,237 proteins. With E-value < 1 × 10^−5^ and max_target_seqs = 5, the retained input comprised 282,859 hits; the MCScanX GFF contained 79,723 gene records. For each cross-species analysis, the longest mapped protein isoform per gene was retained, species prefixes were added to protein and GFF identifiers, and separate Ha + At, Ha + Gm and Ha + Rice datasets were constructed. All-vs-all BLASTP and MCScanX were run independently within each two-species dataset using the same BLASTP thresholds; other MCScanX parameters were kept at default values. Cross-species synteny was plotted using physical chromosome coordinates from both species.

Tandem duplicates and collinearity blocks were identified with MCScanX. Genome-wide duplicate classes were assigned with duplicate_gene_classifier as singleton, dispersed, proximal, tandem or whole-genome duplication/segmental duplication (WGD/segmental). The single category returned by the classifier was used as each gene’s primary duplication class, whereas independently supported overlapping tandem, proximal and WGD/segmental evidence was retained in Table S2. Direct cross-species anchors were assessed separately from intraspecific duplication evidence.

### 4.7. Ka/Ks Selection-Pressure Analysis

Candidate gene pairs were selected using two strategies: MCScanX-supported tandem or recent duplication evidence, and direct Ha-Ha sister pairs with UFBoot ≥ 70 in the intraspecific tree. The final supplementary table retained 13 candidate comparisons, including four primary duplicate comparisons and nine exploratory sister-pair comparisons. Protein sequences were aligned with MAFFT v7.525 using --auto. A custom codon back-translation procedure traversed each protein alignment, consumed the next three coding-sequence nucleotides for every non-gap amino acid and inserted three gap characters (‘---’) at protein-gap positions. Coding-sequence length modulo 3, internal stop codons, ambiguous bases and valid codon counts were checked; no frameshifts, internal stops or ambiguous bases were detected in the retained alignments. PAML v4.10.9 yn00 [50] was run using the standard genetic code (icode = 0, weighting = 0 and commonf3x4 = 0), and Li–Pamilo–Bianchi 1993 (LPB93) estimates of nonsynonymous and synonymous substitution rates (*d*_N_ and d_S_) and their ratio (*ω* = *d*_N_/*d*_S_, also referred to here as Ka/Ks) were reported. All 13 preselected comparisons are provided in Table S4.

### 4.8. Promoter Cis-Regulatory Element Analysis

For each gene, the 2000 bp region upstream of the GFF3-annotated gene boundary was extracted in a strand-aware manner: the interval from gene start − 2000 to gene start − 1 was used for positive-strand genes, whereas the interval from gene end + 1 to gene end + 2000 was reverse-complemented for negative-strand genes. The resulting sequences were submitted to PlantCARE [51] for cis-regulatory element prediction. Interpretable PlantCARE records were assigned to eight functional categories: abscisic acid (ABA), methyl jasmonate (MeJA), salicylic acid (SA), gibberellin (GA), auxin, stress, light and development. Core promoter elements such as TATA-box and CAAT-box and records that could not be stably assigned to the eight categories were retained in the detailed table but excluded from the eight-category visualization summary. For each gene, the per-gene count in each category was used as the variable. Element abundance was compared between Clade II genes and other genes (Clade I plus Other lineages) using the Mann–Whitney *U* test, followed by Benjamini–Hochberg false discovery rate (FDR) correction across the eight categories. Record-level element annotations and category assignments are provided in Table S5a; category-level statistical results are provided in Table S5b.

### 4.9. Transcriptome Expression Analysis

The multidimensional expression analysis covered abiotic stress, biotic stress, hormone treatments and organ/tissue samples. Abiotic stress datasets included combined saline–alkali stress (CRA039257; tolerant K-27 and sensitive K-7, 0, 3, 12, 24, 48 and 96 h) [6], drought stress (SRP355134; drought-tolerant DT and sensitive DS, 0, 7, 14 and 21 d) [52], alkaline stress (SRP294448; leaf and root) [3], and polyethylene glycol (PEG) osmotic stress and salt stress (SRP092742) [30]. Biotic stress datasets included broomrape infestation (SRP382412; resistant R and susceptible S, 0, 4, 12, 24, 48 and 72 h) [4] and *Sclerotinia sclerotiorum* infection (SRP219154; HA89, HA853 and RK416, representing susceptible, moderately tolerant and more tolerant genotypes, respectively, at 0, 4 and 8 days post inoculation) [5]. Hormone treatments included ABA, brassinosteroids (BL), kinetin (KIN), 1-aminocyclopropane-1-carboxylic acid (ACC), GA, indole-3-acetic acid (IAA), methyl jasmonate (MeJA), SA and strigolactone (SL) in leaves and roots from SRP092742. Organ/tissue samples were obtained from SRP092742.

Read quality was assessed using FastQC v0.12.1 [53]. Libraries requiring processing underwent adapter removal and quality filtering with fastp v0.22.0 [54], using the Illumina adapter prefix AGATCGGAAGAGC or automatic adapter detection for paired-end data. Reads with more than 40% low-quality bases (Q < 15), more than five N bases or a length below 20 nt were removed. For libraries requiring tail trimming, a 4 nt window and a mean quality threshold of Q20 were used. Reads were aligned to a HISAT2 v2.2.2 [55] index built from the HanXRQr2.0-SUNRISE genomic FASTA in the corresponding single- or paired-end mode. Gene-level counts were summarized with featureCounts v2.0.6 [56] using exon features and gene_id grouping (-t exon -g gene_id). Paired-end libraries were processed with -p --countReadPairs -B to count fragments, including only fragments with both ends successfully aligned; single-end libraries were counted as individual reads. Strandedness was assessed for each library using infer_experiment.py from RSeQC v5.0.2 [57]. Libraries with balanced orientation were counted as unstranded (-s 0), whereas those showing a reverse-stranded pattern were counted as reverse-stranded (-s 2). Multimapping reads or fragments and those overlapping more than one gene were excluded from counting. Gene-level transcripts per million (TPM) values were calculated from featureCounts-derived gene lengths.

The final expression matrix comprised 200 included libraries summarized into 87 distinct conditions. Of these conditions, 30 were represented by one included library, 11 by two, 36 by three and 10 by four. TPM values were averaged across included libraries for conditions with multiple libraries, whereas single-library conditions were retained as individual observations. An overall alignment rate of at least 50% was required for inclusion, and one additional library with extremely low sequencing depth was excluded. Per-library read counts, overall alignment rates, featureCounts gene-assignment rates and inclusion status are listed in Table S6b. log_2_(TPM + 1) values were used for manuscript descriptions and heatmap plotting without row scaling. The heatmap display range was 0–4, with values above 4 shown using the highest color level. A breadth threshold of log_2_(TPM + 1) > 3 was used only for descriptive display and corresponds approximately to TPM > 7. For group-level descriptive summaries, an unweighted grand mean was calculated across all gene-condition log_2_(TPM + 1) values assigned to each phylogenetic group, duplication group or within-clade duplication group; these cross-project summaries were not used as inferential group comparisons. Comparisons across study sources and summary methods, and sensitivity analyses of breadth thresholds and member or source inclusion, are detailed in the Supplementary Robustness Analyses (Tables S8–S12). Sample metadata and library-type annotations are provided in Table S6a, and TPM values for the 33 genes across the 87 distinct conditions are provided in Table S6c.

Integer counts for all genes in CRA039257 were analyzed using DESeq2 v1.50.2 [58], with genotype, categorical time and their interaction included to assess baseline differences and changes over time. Individual contrasts were evaluated with Wald tests, and the overall interaction was evaluated with a likelihood-ratio test. Benjamini–Hochberg correction was applied separately across all testable genes for each contrast or overall interaction test, with an adjusted *p* value < 0.05 as the significance threshold. Per-library expression values and replicate variation are provided in Table S6d; effect sizes and unadjusted 95% confidence intervals for HaSABATH genes are listed in Table S6e; the remaining public transcriptomes were used for descriptive expression analysis.

### 4.10. Protein Three-Dimensional Structure Prediction and Confidence Analysis

ColabFold v1.5.2 [59,60] was used to predict AlphaFold2 structures for the 33 HaSABATH proteins. The predictions used the alphafold2_ptm model type and mmseqs2_uniref_env multiple sequence alignment (MSA) mode. Five candidate models were generated for each protein (models 1–5, seed 000) and ranked by predicted template modeling score (pTM). The rank-1 unrelaxed model selected by pTM was used for downstream analysis.

Residue-level predicted local distance difference test (pLDDT) values were extracted from the B-factor field of Cα atoms in the AlphaFold2 Protein Data Bank-formatted files. These values were used to calculate mean pLDDT, the proportion of low-confidence residues and residue confidence composition. The rank-1 score file in JavaScript Object Notation format was used to extract pTM and maximum predicted aligned error (PAE). Eight representative proteins (HaSABATH4, HaSABATH5, HaSABATH6, HaSABATH24, HaSABATH15, HaSABATH16, HaSABATH22 and HaSABATH31) were selected to cover the three phylogenetic groups, major duplication contexts, expression-prioritized candidates and a range of model-confidence values; HaSABATH24 was retained as the lower-confidence comparison. The models were rendered individually as pLDDT-colored ribbons in ChimeraX [61], and length scale bars were added according to the display scale of each view.

## 5. Conclusions

This study establishes a high-confidence framework for the sunflower *HaSABATH* family by integrating phylogenetic classification, duplication history, cross-species synteny, promoter architecture, structure-prediction confidence and multidimensional transcriptome profiles.

Local duplication and WGD/segmental duplication jointly shaped the family, with overlapping duplication evidence retained for *HaSABATH4* and *HaSABATH15*. The limited number of direct cross-species *HaSABATH* anchors indicates that detectable syntenic conservation is concentrated in a subset of genes, whereas the 11 reliable Ka/Ks estimates below 1 support predominant purifying constraint among the analyzed pairs. Most predicted structures had high overall confidence and were consistent with the expected SABATH architecture, although these models do not establish substrate specificity or enzyme activity.

Across the sampled conditions, Clade I members generally showed higher expression levels and greater expression breadth, whereas many Clade II members were weakly expressed or condition-biased. Integrated evidence prioritizes *HaSABATH4* and *HaSABATH6* for follow-up under combined saline–alkali stress; *HaSABATH31*, which was broadly expressed, and *HaSABATH16*, which was highly expressed in the capitulum, also warrant further study. These resources provide a basis for future genetic and biochemical validation of SABATH functions in sunflower stress adaptation and secondary metabolism.

## Figures and Tables

**Table 1:** Basic characteristics and primary duplication types of 33 *HaSABATH* genes.

Name	Gene ID	Chr	Clade	Length (aa)	MW (kDa)	pI	CDS Exons	Duplication
*HaSABATH1*	*LOC110902710*	Chr02	Clade I	363	40.95	6.17	4	Tandem
*HaSABATH2*	*LOC110897955*	Chr02	Clade I	363	40.81	6.22	4	Tandem
*HaSABATH3*	*LOC110923123*	Chr02	Clade II	369	41.30	5.95	4	Dispersed
*HaSABATH4*	*LOC110899290*	Chr02	Clade II	359	41.04	5.95	5	WGD/Seg.
*HaSABATH5*	*LOC110926259*	Chr02	Clade II	370	42.30	5.76	4	Tandem
*HaSABATH6*	*LOC110926270*	Chr02	Clade II	359	41.14	6.08	4	Proximal
*HaSABATH7*	*LOC110890009*	Chr04	Clade II	371	41.87	6.53	4	Dispersed
*HaSABATH8*	*LOC110937826*	Chr04	Clade II	369	41.98	6.32	4	Dispersed
*HaSABATH9*	*LOC110864671*	Chr06	Clade II	353	40.22	7.14	3	Dispersed
*HaSABATH10*	*LOC110944463*	Chr06	Other lineages	361	40.12	5.77	3	Dispersed
*HaSABATH11*	*LOC110878856*	Chr09	Clade I	367	41.29	5.14	4	WGD/Seg.
*HaSABATH12*	*LOC110878855*	Chr09	Clade I	367	41.29	5.33	5	Proximal
*HaSABATH13*	*LOC110878878*	Chr09	Clade I	367	41.35	5.14	4	WGD/Seg.
*HaSABATH14*	*LOC110878877*	Chr09	Clade I	369	41.66	5.19	4	Tandem
*HaSABATH15*	*LOC110878876*	Chr09	Clade I	369	41.61	5.12	4	WGD/Seg.
*HaSABATH16*	*LOC110889462*	Chr11	Clade I	360	40.73	5.78	4	WGD/Seg.
*HaSABATH17*	*LOC110889463*	Chr11	Clade I	368	41.75	5.20	4	Proximal
*HaSABATH18*	*LOC110889848*	Chr11	Clade II	367	41.22	6.26	4	Proximal
*HaSABATH19*	*LOC110889847*	Chr11	Clade II	368	41.60	5.89	4	Proximal
*HaSABATH20*	*LOC110889846*	Chr11	Clade II	368	41.10	6.22	4	Proximal
*HaSABATH21*	*LOC110889844*	Chr11	Clade II	368	41.14	6.42	4	Proximal
*HaSABATH22*	*LOC110890061*	Chr11	Other lineages	390	42.55	5.89	4	Dispersed
*HaSABATH23*	*LOC110894917*	Chr12	Clade II	352	39.75	8.11	4	Dispersed
*HaSABATH24*	*LOC110902813*	Chr13	Clade II	459	52.09	6.07	4	WGD/Seg.
*HaSABATH25*	*LOC110898159*	Chr13	Clade II	388	44.37	5.38	4	Tandem
*HaSABATH26*	*LOC110898160*	Chr13	Clade II	388	44.37	5.38	4	Tandem
*HaSABATH27*	*LOC110873171*	Chr15	Clade II	371	41.74	6.70	4	Proximal
*HaSABATH28*	*LOC110926806*	Chr15	Clade II	371	41.72	6.64	4	Proximal
*HaSABATH29*	*LOC110911355*	Chr15	Clade II	371	41.76	6.46	4	Proximal
*HaSABATH30*	*LOC110911354*	Chr15	Clade II	371	41.80	6.64	4	Proximal
*HaSABATH31*	*LOC110915341*	Chr16	Clade I	360	41.34	5.55	4	WGD/Seg.
*HaSABATH32*	*LOC110915347*	Chr16	Clade I	379	42.34	5.31	4	WGD/Seg.
*HaSABATH33*	*LOC110923156*	Chr17	Clade I	363	41.23	5.93	4	Dispersed

Abbreviations: Chr, chromosome; aa, amino acids; MW, molecular weight; pI, isoelectric point; CDS, coding sequence; GRAVY, grand average of hydropathicity; WGD, whole-genome duplication; Seg., segmental duplication. Note: The exon column indicates CDS exon count. Duplication type indicates the nonredundant primary duplication category. *HaSABATH4* and *HaSABATH15* are counted as WGD/Segmental in the primary category but also have tandem evidence. Full LOC IDs, coordinates, gene exon counts, CDS exon counts, gene spans, CDS lengths, GRAVY values, domain coordinates, BLASTP best hits and overlapping duplication evidence are provided in Table S2.

**Table 2:** **Direct cross-species syntenic anchors involving *HaSABATH* genes**.

Comparison	Ha Gene	Reference Partner ID	BLASTP E-Value	Interpretation
Ha-At	*HaSABATH14*	*AT4G36470*	2 × 10^−94^	Ha-At-specific direct anchor
Ha-At	*HaSABATH22*	*AT5G55250*	0	Shared dicot-comparison anchor
Ha-At	*HaSABATH31*	*AT4G36470*	3 × 10^−153^	Shared dicot-comparison anchor
Ha-Gm	*HaSABATH22*	*LOC100805902*	0	Shared dicot-comparison anchor
Ha-Gm	*HaSABATH31*	*LOC100814233*	5 × 10^−99^	Shared dicot-comparison anchor
Ha-Rice	None	None	-	No direct *HaSABATH* anchor detected

## Data Availability

No new sequencing data were generated specifically for this study. The sunflower HanXRQr2.0-SUNRISE genome (GCF_002127325.2), *Arabidopsis* TAIR10 genome (GCF_000001735.4), soybean Wm82.a4.v1 genome and rice IRGSP-1.0 genome are publicly available from NCBI or Phytozome. The public transcriptome datasets used here are CRA039257, SRP355134, SRP294448, SRP092742, SRP382412 and SRP219154. The supplementary workbook contains reference-sequence metadata (Table S1), detailed *HaSABATH* gene features (Table S2), motif summaries, occurrences and position weight matrices (Table S3a–c), Ka/Ks results (Table S4), record-level cis-regulatory element annotations and category statistics (Table S5a,b), transcriptome sample metadata (Table S6a), per-library quality-control results (Table S6b), the matrix of mean TPM values for each condition (Table S6c), replicate expression values and variation summaries (Table S6d), within-project statistical comparisons (Table S6e), and structure-prediction confidence metrics (Table S7). Supplementary Sequence Data contains the four supplementary FASTA datasets. Additional robustness methods and results are provided in Additional File S4 (Tables S8–S13).

## Supplementary Materials

The following supporting information can be downloaded at: https://www.mdpi.com/article/10.3390/ijms27219805/s1. The Supplementary Materials cite references [3,4,5,6,8,9,13,14,15,16,17,20,30,42,45,47,52,58,62,63,64,65,66,67,68].

## Abbreviations

aa	Amino acid
ABA	Abscisic acid
ABRE	ABA-responsive element
ACC	1-Aminocyclopropane-1-carboxylic acid
BA	Benzoic acid
BAMT	Benzoic acid carboxyl methyltransferase
BL	Brassinosteroids
BLASTP	Protein BLAST
BSMT	Benzoic acid/salicylic acid carboxyl methyltransferase
CDS	Coding sequence
Ck	Control
CLAMT	Carlactonoic acid methyltransferase
*d*_N_	Nonsynonymous substitution rate
*d*_N_/*d*_S_	Nonsynonymous/synonymous substitution-rate ratio (also referred to here as Ka/Ks)
dpi	Days post inoculation
*d*_S_	Synonymous substitution rate
FDR	False discovery rate
GA	Gibberellin
GFF3	General Feature Format version 3
GRAVY	Grand average of hydropathicity
HMM	Hidden Markov model
HMMER	Hidden-Markov-model-based profile search software
IAA	Indole-3-acetic acid
IAMT	Indole-3-acetic acid carboxyl methyltransferase
JA	Jasmonic acid
JMT	Jasmonic acid carboxyl methyltransferase
KIN	Kinetin
LPB93	Li–Pamilo–Bianchi 1993 method
MAFFT	Multiple alignment using fast Fourier transform
MCScanX	Multiple Collinearity Scan toolkit X
MeJA	Methyl jasmonate
MEME	Multiple EM for motif elicitation
MeSA	Methyl salicylate
ML	Maximum likelihood
MSA	Multiple sequence alignment
MW	Molecular weight
MXMT	7-Methylxanthine methyltransferase
NAMT	Nicotinic acid methyltransferase
NCBI	National Center for Biotechnology Information
PAE	Predicted aligned error
PAML	Phylogenetic analysis by maximum likelihood
PEG	Polyethylene glycol
pI	Isoelectric point
pLDDT	Predicted local distance difference test
pTM	Predicted template modeling score
qRT-PCR	Quantitative reverse-transcription PCR
RNA-seq	RNA sequencing
SA	Salicylic acid
SABATH	Salicylic acid carboxyl methyltransferase, benzoic acid carboxyl methyltransferase and theobromine synthase
SAM	S-adenosyl-L-methionine
SAMT	Salicylic acid carboxyl methyltransferase
SL	Strigolactone
TAIR	The *Arabidopsis* Information Resource
TPM	Transcripts per million
UFBoot	Ultrafast bootstrap
UTR	Untranslated region
WGD	Whole-genome duplication
XMT/DXMT	Xanthosine methyltransferase/dimethylxanthine methyltransferase
