# Supplementary material for: Genome-Wide Characterization and Multidimensional Transcriptome Profiling of the SABATH Methyltransferase Gene Family in Sunflower (Helianthus annuus L.)
Source: Int J Mol Sci. 2026 Oct 31;27(21):9805. doi: 10.3390/ijms27219805 (PMC13763386; doi:10.3390/ijms27219805)
Supplement: Supplementary file 1 [file ijms-27-09805-s001.zip › Additional_file_S2_Supplementary_Figures_S1-S2.pdf]

Additional file 2. Supplementary Figures S1-S2

Figure S1. Complete phylogenetic tree of 148 SABATH proteins

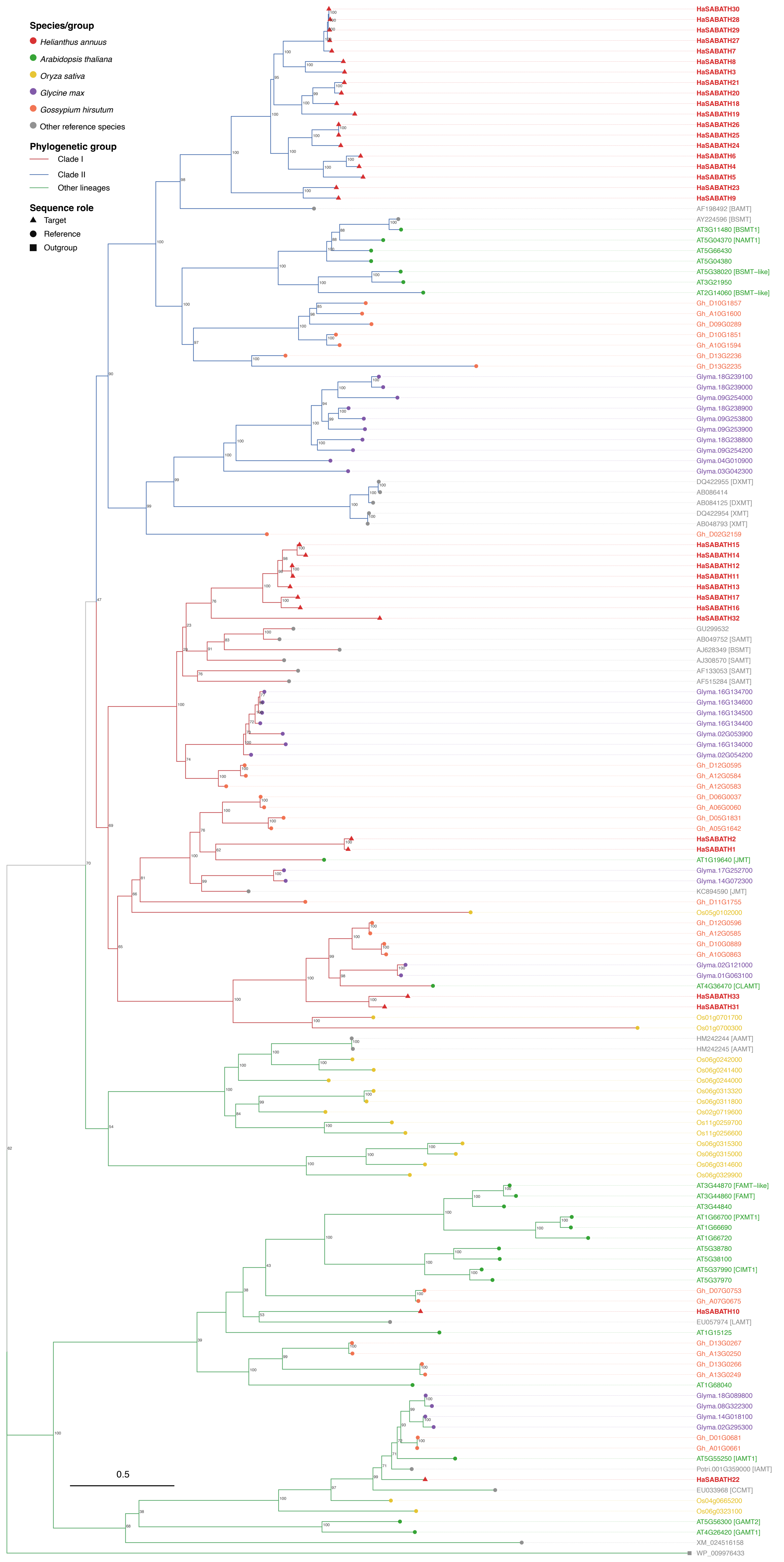

Figure S1. Complete multi-species phylogeny of SABATH proteins.
The maximum-likelihood tree includes 33 HaSABATH proteins and 115 reference SABATH sequences, with the bacterial Methyltransf_7 homolog WP_009976433 as the outgroup. Tip labels and branch colours indicate species and phylogenetic group annotations. The tree provides full tip labels corresponding to the condensed phylogenetic classification used in Figure 1.

Figure S2. MEME motif logos reconstructed from PWM

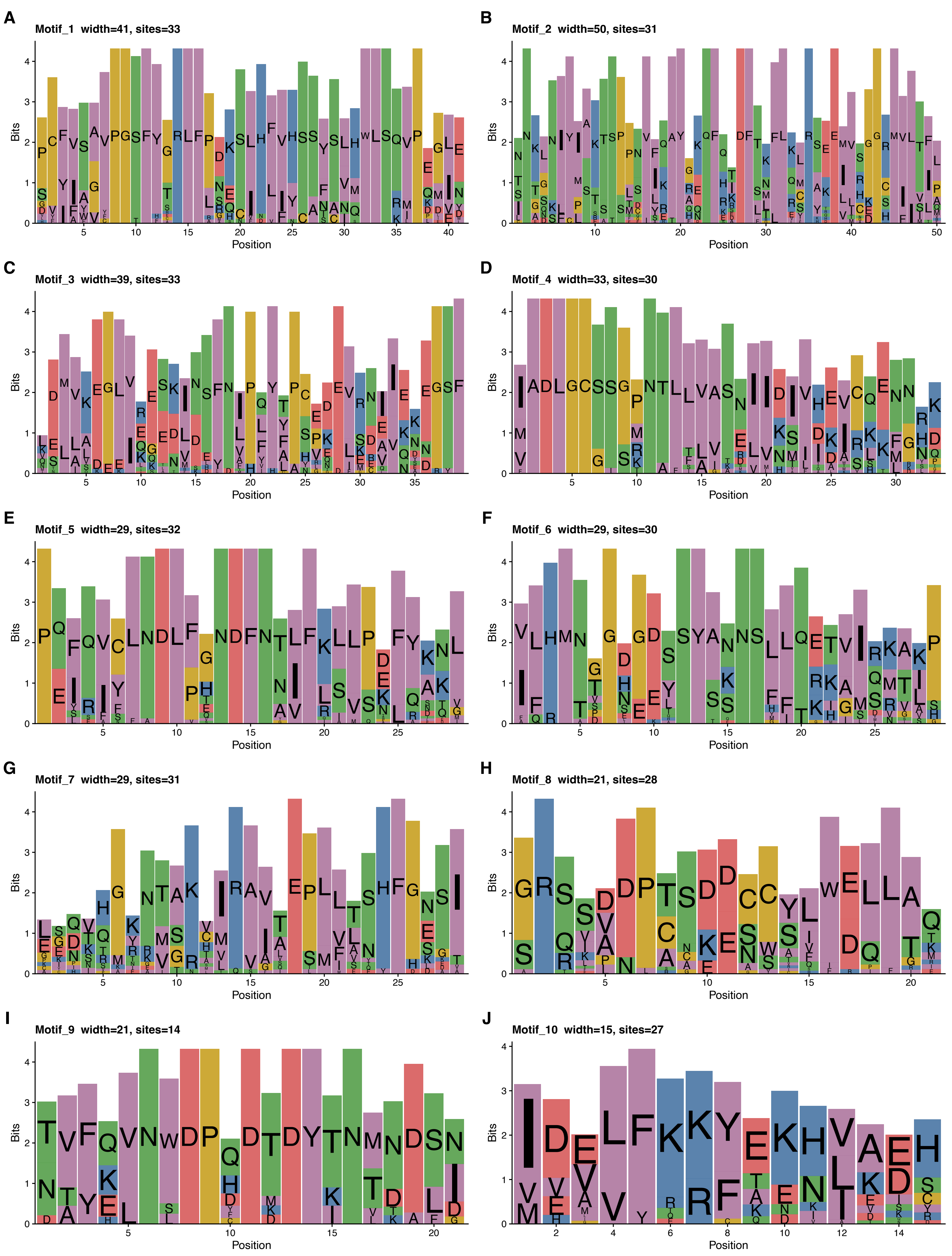

Figure S2. MEME motif logos for the 10 conserved HaSABATH protein motifs.
The composite logo panel shows sequence logos reconstructed from MEME position-weight matrices for the 10 conserved motifs identified in the 33 HaSABATH proteins. Motif identifiers correspond to the motif-occurrence and PWM records in Supplementary Table S3.
