# Supplementary material for: Genome-Wide Characterization and Multidimensional Transcriptome Profiling of the SABATH Methyltransferase Gene Family in Sunflower (Helianthus annuus L.)
Source: Int J Mol Sci. 2026 Oct 31;27(21):9805. doi: 10.3390/ijms27219805 (PMC13763386; doi:10.3390/ijms27219805)
Supplement: Supplementary file 1 [file ijms-27-09805-s001.zip › Additional_file_S4_Supplementary_Robustness_Analyses.pdf]

Robustness analyses of sunflower SABATH genes

Supplementary methods and results
Tables S8–S13

Expression profiles from six study sources were integrated to describe 33 sunflower SABATH members, alongside multispecies phylogenetic analyses of their lineage relationships. These supplementary analyses examine study composition, expression-breadth thresholds, member inclusion and outgroup choice to identify consistent patterns and their scope across settings.

1 Effects of study composition and summary method

Combining studies broadens the range of organs and treatments examined, but the six study sources contributed different numbers of conditions, ranging from 4 to 33. Sources with more conditions contribute more to a summary pooled across conditions. We therefore compared expression within each source and then used two summary methods to assess whether the overall direction depended on source contributions.

The expression data included 200 retained libraries, 87 distinct conditions and 33 HaSABATH members (Tables S6a–S6c). For each gene, TPM values from the included libraries were averaged within each condition and transformed as $\log_2$(mean TPM + 1). Mean log expression denotes the arithmetic mean of these transformed values, before application of the heatmap display cap. The shared leaf and root controls are each shown twice in Figure 6, giving 89 displayed entries representing 87 distinct conditions.

Clades I and II contained 12 and 19 sunflower members, respectively; the other two members belonged to other lineages. Grouping followed the primary duplication categories in Table S2. Members assigned to whole-genome duplication (WGD)/segmental duplication are termed the WGD group here; all other primary categories form the non-WGD group. The WGD/non-WGD member counts were 6/6 in Clade I and 2/17 in Clade II. Comparisons within individual study sources are summarized in Table S8.

Table S8. Mean log expression by clade and duplication category within six study sources

Source	Distinct conditions	Clade I / II<br>Mean log expression	Within Clade I<br>WGD / non-WGD	Within Clade II<br>WGD / non-WGD
CRA039257	12	1.8918 / 0.6833	2.1059 / 1.6778	0.6288 / 0.6897
SRP092742	33	1.1392 / 0.3843	1.6701 / 0.6083	0.0924 / 0.4186
SRP219154	18	2.1144 / 1.0245	3.0436 / 1.1851	0.8441 / 1.0458
SRP294448	4	1.7852 / 0.8606	1.9956 / 1.5748	1.6208 / 0.7711
SRP355134	8	1.5258 / 0.8793	1.7294 / 1.3223	1.3705 / 0.8215
SRP382412	12	1.7705 / 0.8045	2.5041 / 1.0368	0.2501 / 0.8698

Note. Paired values follow the order in the column headings and are arithmetic means of $\log_2$(mean TPM + 1) for each group. Within each source, each gene–condition value contributes equally to the group mean.

Under “Pooled conditions”, transformed expression values are averaged across group members and all 87 conditions, with breadth calculated over the same combinations. For “Mean across sources”, each metric is calculated within each source before taking the arithmetic mean of the six summaries (Table S9). Expression breadth uses the primary threshold $\log_2$(mean TPM + 1) > 3; its definition and alternative thresholds are described in Section 2.

Table S9. Within-clade WGD and non-WGD expression under two summary methods

Clade	Members WGD / non-WGD	Summary method	Mean log expression WGD / non-WGD	Breadth (> 3) WGD / non-WGD
Clade I	6 / 6	Pooled conditions	2.1498 / 1.0443	27.20% / 13.22%
Clade I	6 / 6	Mean across sources	2.1748 / 1.2342	27.37% / 16.46%
Clade II	2 / 17	Pooled conditions	0.5315 / 0.7012	6.32% / 8.05%
Clade II	2 / 17	Mean across sources	0.8011 / 0.7694	11.11% / 9.52%

Note. Paired values represent the WGD and non-WGD groups, respectively. Expression breadth uses $\log_2$(mean TPM + 1) > 3; summary methods are defined in the accompanying methods.

Within all six sources, Clade I had higher mean log expression than Clade II (Table S8). The Clade I WGD group also had higher mean log expression than the non-WGD group in every source. Under both summary methods, the Clade I WGD group had higher mean log expression and greater expression breadth at the primary threshold. The direction of the Clade II comparison changed between summary methods (Table S9). The "Mean across sources" summary gave breadths of 21.91% and 9.69% for Clades I and II at the primary threshold, matching the pooled direction.

2 Effects of expression-breadth thresholds and member inclusion

Expression breadth summarizes continuous expression as the proportion exceeding a threshold, making the metric sensitive to values near that threshold. Group means can also be influenced by individual members or study sources. We varied the thresholds and omitted members or sources in turn to assess whether the main patterns persisted.

Expression breadth was the proportion of gene–condition combinations exceeding a specified threshold among all combinations in the group. We compared $\log_2$(mean TPM + 1) > 2, > 3 (the primary threshold) and > 4, corresponding to mean TPM > 3, > 7 and > 15. Across the 87 conditions, Clade I had greater breadth than Clade II at all three thresholds. The overall WGD group also had greater breadth than the non-WGD group (Table S10). The WGD/non-WGD comparison across the entire family also reflects clade composition; within-clade results are provided in Table S9.

Table S10. Group summaries across three expression-breadth thresholds

Group	Mean log expression	Breadth > 2	Breadth > 3	Breadth > 4
Clade I	1.5971	31.80%	20.21%	13.41%
Clade II	0.6834	13.19%	7.86%	3.81%
All WGD/segmental members	1.7452	35.06%	21.98%	15.23%
All non-WGD members	0.7941	15.45%	9.61%	4.83%

Note. Values summarize all 87 conditions. Clade I, Clade II, all WGD members and all non-WGD members contain 12, 19, 8 and 25 genes, respectively. Expression-breadth denominators are the group member count × 87. The last two rows cover the entire family by duplication category, overlapping the clade classification in the first two rows.

Of the 18 combinations of six sources and three thresholds, 14 showed greater breadth in the Clade I WGD group, matching the pooled direction. Four sources retained this direction at all three thresholds, and all six sources did so at > 2. Three combinations had lower WGD breadth and one had equal breadth, occurring in SRP294448 and SRP355134 at > 3 or > 4 (Table S11).

Table S11. Expression breadth within Clade I by source and threshold

Source	> 2<br>WGD / non-WGD	> 3<br>WGD / non-WGD	> 4<br>WGD / non-WGD
CRA039257	50.00% / 36.11%	31.94% / 23.61%	20.83% / 11.11%
SRP092742	34.85% / 9.60%	17.68% / 7.07%	9.60% / 3.03%
SRP219154	47.22% / 19.44%	38.89% / 16.67%	35.19% / 13.89%
SRP294448	41.67% / 29.17%	16.67% / 25.00%	16.67% / 16.67%
SRP355134	43.75% / 35.42%	18.75% / 20.83%	4.17% / 10.42%
SRP382412	52.78% / 23.61%	40.28% / 5.56%	33.33% / 0.00%

Note. Thresholds apply to $\log_2$(mean TPM + 1). Paired values represent WGD and non-WGD expression breadth, respectively. Each group contains six members, so each denominator is 6 × the number of conditions in that source. Directions were determined from unrounded proportions.

HaSABATH25 and *HaSABATH26* have identical annotated exon sequences, and their member-specific expression estimates are limited by ambiguity in read assignment. We omitted their expression-matrix rows and recalculated summaries to assess how their inclusion affected group comparisons. We also omitted one Clade I member or one source at a time to assess whether the WGD–non-WGD mean difference depended on individual contributions (Table S12). These checks used the existing TPM matrix, retaining the TPM values of all remaining members.

Table S12. Within-clade WGD–non-WGD mean log expression differences after member or source omission

Omission	Clade	Summary method	Baseline difference	Difference after omission or its range
Omit *Ha25* and *Ha26*	Clade II	Pooled conditions	-0.1698	-0.2542
Omit *Ha25* and *Ha26*	Clade II	Mean across sources	0.0317	-0.0614
Omit one member (12 cases)	Clade I	Pooled conditions	1.1055	0.7134 to 1.4527
Omit one member (12 cases)	Clade I	Mean across sources	0.9406	0.5648 to 1.2872
Omit one source (6 cases)	Clade I	Pooled conditions	1.1055	0.9090 to 1.2139
Omit one source (6 cases)	Clade I	Mean across sources	0.9406	0.7570 to 1.0473

Note. Differences are WGD minus non-WGD mean log expression, calculated before rounding. *Ha25* and *Ha26* denote *HaSABATH25* and *HaSABATH26*, respectively. Omitting both changes Clade II WGD/non-WGD member counts from 2/17 to 2/15. For each single omission, summaries were recalculated from the remaining data; ranges are the minimum and maximum differences obtained.

The Clade I mean log expression difference remained positive under both summary methods after each member or source omission. In Clade II, the difference under "Mean across sources" changed from 0.0317 to −0.0614 after omitting *HaSABATH25* and *HaSABATH26*. Its direction therefore changed with the inclusion of these members. The two Clade II WGD members, *HaSABATH4* and *HaSABATH24*, correspond to one segmental duplication relationship. All expression comparisons above are descriptive summaries within the current sampling scope.

3 Sensitivity to outgroup choice

Figure 1 used the distant homolog MaSABATH as an outgroup reference. We first examined the comparable information it shared with plant sequences in the original alignment. We then varied outgroups to examine relationships among sunflower members and broader multispecies branches separately. Fungal and green algal references, individually and together, provided eukaryotic homologs from different sources.

In the 793-column alignment used for Figure 1, plant core columns were defined by standard amino acid residues in at least 80% of the 147 plant sequences. This criterion identified 345 plant core columns in the original alignment. MaSABATH occupied 320 of these columns, giving an occupancy of 92.75%. This percentage measures residue occupancy, not sequence identity or alignment accuracy. Its gap percentage across the full alignment was 55.23%, close to the median of 53.47% for the 147 plant sequences.

Keeping the 147 plant sequences and their 793 alignment coordinates fixed, we compared five outgroup settings. These used a bacterial outgroup, no outgroup, a fungal outgroup, a green algal outgroup, or combined fungal and green algal outgroups. The bacterial reference was MaSABATH from *Mycobacterium avium* (WP_009976433). Following the eukaryotic SABATH outgroups used by Han et al. [20], we selected homologs from *Aspergillus clavatus* (XP_001270502.1) and *Coccomyxa subellipsoidea* (XP_005646815.1), a green alga.

MAFFT v7.526 added the eukaryotic references to the original alignment using --add --keeplength --mapout [42]. Original plant sequence rows and column coordinates were preserved, and the required sequences were selected for each setting. Trees were reconstructed separately in IQ-TREE v2.3.6 using the Q.plant+R5 model form, with parameters estimated for each tree [45]. Each setting was analyzed once with 1,000 ultrafast bootstrap (UFBoot) replicates [47]. Seeds 26101301–26101305 were used successively in the setting order shown in Table S13.

Trees were pruned to the shared set of 33 HaSABATH members or 147 plant sequences to compare nontrivial unrooted splits and Robinson–Foulds (RF) distances. The original multispecies Clades I and II in Table S13 contain 47 and 51 sequences, respectively, including references. These sets differ from the 12 and 19 sunflower members in the expression analysis.

Table S13. Five outgroup settings with fixed plant alignment coordinates and a common model form

Outgroup setting	Total sequences	33 HaSABATH members RF / shared splits	147 plant sequences RF	Original multispecies sets Clade I / II UFBoot
Bacterial outgroup	148	0 / 30	0	70 / 92
No outgroup	147	0 / 30	12	70 / 88
Fungal outgroup	148	0 / 30	4	64 / 90
Green algal outgroup	148	0 / 30	20	Not retained / Not retained
Fungal + green algal outgroups	149	0 / 30	12	46 / 78

Note. RF is the unnormalized Robinson–Foulds distance from the original Figure 1 tree on the shared leaf set. A distance of 0 indicates identical unrooted topology for that leaf set; it does not measure rooting confidence. There are 30 nontrivial unrooted splits among the 33 HaSABATH members. The final column evaluates the exact original multispecies Clade I and II sets, containing 47 and 51 sequences, respectively. Values are UFBoot support (0–100) for the corresponding internal edge in the tree reconstructed under each setting. “Not retained” indicates that the set did not form the corresponding split.

All five settings retained all 30 nontrivial unrooted splits among the 33 HaSABATH members, with RF = 0 relative to the original tree. RF distances for the 147 plant sequences ranged from 0 to 20 across the settings. The green algal outgroup did not retain the splits corresponding to the exact original Clade I and II membership sets. The combined outgroup retained both sets, with UFBoot support of 46 and 78, respectively (Table S13).

Terminal branch length describes the divergence of an outgroup sequence in a reconstructed tree. It measures expected substitutions per amino acid site between the sequence and its adjacent internal node. In these control reconstructions, the fungal homolog used by Han et al. [20] had a terminal branch length of 3.7195, versus 3.2770 for the bacterial control. Replacing the bacterial reference with this fungal sequence therefore did not shorten the outgroup terminal branch. In this dataset, a eukaryotic origin did not necessarily correspond to a shorter terminal branch. Branch lengths should be interpreted together with comparable sites and topology results.

MaSABATH occupancy of plant core columns and its gap percentage provide a sequence-level basis for comparison using this distant homologous reference. Across these five reconstructions, the unrooted topology among sunflower members remained unchanged, whereas some multispecies branches differed.

References

Reference numbers follow the main manuscript.

20. Han XM, Yang Q, Liu YJ, Yang ZL, Wang XR, Zeng QY, et al. Evolution and function of the *Populus* SABATH family reveal that a single amino acid change results in a substrate switch. Plant Cell Physiol. 2018;59:392–403. https://doi.org/10.1093/pcp/pcx198.

42. Katoh K, Standley DM. MAFFT multiple sequence alignment software version 7: improvements in performance and usability. Mol Biol Evol. 2013;30:772–780. https://doi.org/10.1093/molbev/mst010.

45. Minh BQ, Schmidt HA, Chernomor O, Schrempf D, Woodhams MD, von Haeseler A, et al. IQ-TREE 2: new models and efficient methods for phylogenetic inference in the genomic era. Mol Biol Evol. 2020;37:1530–1534. https://doi.org/10.1093/molbev/msaa015.

47. Hoang DT, Chernomor O, von Haeseler A, Minh BQ, Vinh LS. UFBoot2: improving the ultrafast bootstrap approximation. Mol Biol Evol. 2018;35:518–522. https://doi.org/10.1093/molbev/msx281.
